# Modulation of cytotoxic amyloid fibrillation and mitochondrial damage of α-synuclein by catechols mediated conformational changes

**DOI:** 10.1038/s41598-023-32075-9

**Published:** 2023-03-31

**Authors:** Toktam Zohoorian-Abootorabi, Ali Akbar Meratan, Saeed Jafarkhani, Vladimir Muronetz, Thomas Haertlé, Ali Akbar Saboury

**Affiliations:** 1 Institute of Biochemistry and Biophysics, University of Tehran, Tehran, 14176-14335 Iran; 2 Department of Biological Sciences, Institute for Advanced Studies in Basic Sciences (IASBS), Zanjan, 45137-66731 Iran; 3 Division of Biomedical Engineering, Faculty of New Sciences and Technologies, University of Tehran, Tehran, 57131-14399 Iran; 4 Belozersky Institute of Physico-Chemical Biology, Lomonosov Moscow State University, Moscow, 119991 Russia; 5 National Institute of Agronomic and Environmental Research, 44316 Nantes, France

**Keywords:** Biochemistry, Biophysics, Cell biology

## Abstract

The interplay between α-synuclein (α-syn) and catechols plays a central role in Parkinson’s disease. This may be related to the modulating effects of catechols on the various aspects of α-syn fibrillization. Some of these effects may be attributed to the membrane-binding properties of the protein. In this work, we compare the effect of some catechols, including dopamine, epinephrine, DOPAL, and levodopa in micromolar concentrations, on the in vitro cytotoxicity of α-syn fibrils on human neuroblastoma SH-SY5Y cells. The study was followed by comparing the interactions of resulting structures with rat brain mitochondria used as an in vitro biological model. The obtained results demonstrate that catechols-induced structures have lost their cytotoxicity mimicking apoptotic cell death mediated by α-syn aggregates in different proportions. Moreover, α-syn fibrils-induced mitochondrial dysfunction, evaluated by a range of biochemical assays, was modulated by catechols-modified α-syn oligomers in different manners, as levodopa and DOPAL demonstrated the maximal and minimal effects, respectively. The plausible mechanism causing the inhibition of α-syn cytotoxic fibrillization and mitochondrial dysfunction by catechols is discussed. Taken together, we propose that catechols can prevent the cytotoxic assembly of α-syn and its destructive effects on mitochondria at various stages, suggesting that decreased levels of catechols in dopaminergic neurons might accelerate the α-syn cytotoxicity and mitochondrial dysfunction implicating Parkinson’s disease.

## Introduction

Many human diseases are associated with the accumulation of abnormally folded proteins, termed amyloids. Amyloid fibrils are pathological aggregates of proteins misfolded into β-sheet-rich conformation. They are a hallmark of various neurodegenerative disorders, including Parkinson’s and Alzheimer’s diseases^1^. Among them, Parkinson’s disease (PD) is the second common neurodegenerative disease and the most prevalent neurological movement disorder^2^. Clinically, PD is characterized by motor dysfunction that manifests as bradykinesia, rigidity and postural instability. α-synuclein (α-syn) is a 140 amino acid protein that is predominantly localized in the presynaptic terminals of neurons^3,4^. Physiological functions of α-syn are associated with synaptic vesicle trafficking and membrane fusion^5^. α-Syn is intrinsically disordered in solution, adopts an amphipathic helical conformation upon membrane association, and plays a key role in synaptic vesicle docking, fusion, and clustering^6^. While misfolding and deposition of wild-type α-syn in Lewy bodies and Lewy neurites is considered as the main pathological hallmark of PD, mutations in the gene encoding for α-syn are associated with both familial and sporadic forms of PD and found to modulate the aggregation process of protein^7–9^. Although α-syn accounts for as much as 1% of the total protein in soluble cytosolic brain fractions^10^, its deposits are specifically formed in dopaminergic neurons in substantia nigra *pars compacta* (SN*pc*). This may suggest a connection between α-syn aggregation and dopamine metabolism. In this regard, evidence obtained by extensive studies strongly supports the notion that fibrillation of α-syn and dopamine metabolism are closely linked to the pathogenesis of PD^11^. These findings also imply a connection between cytosolic α-syn and dopamine, leading to the formation of adducts stabilized by covalent and non-covalent interactions^12^, suggesting that dopamine and other catechols could be critical modulators of α-syn fibrillation.

The results obtained by extensive in vitro and in vivo studies indicate that catechol derivatives, including dopamine, modulate the assembly process of α-syn by inhibiting the formation of amyloid fibrils, but promoting the accumulation of oligomers/protofibrils species^13^ or by disaggregating of amyloidogenic aggregates^14^. This modulating effect of catechols may be related to conformational changes in α-syn^15^ occurring through the interaction of dopamine metabolites with specific sequences of the protein^16^, which can alter the membrane binding properties of α-syn^17^. Besides misfolding and aggregation of α-syn leading to degeneration of dopaminergic neurons^5^, impairment of mitochondrial function and an associated increase in oxidative stress have been found to be key contributors to the pathogenesis of PD. For instance, cellular overexpression of wild-type and mutant α-syn has been shown to induce mitochondrial dysfunction leading to oxidative stress^18^. Such mitochondrial abnormalities were also observed in A53T transgenic mice as well as in the brain of PD patients^19^.

**Figure 1:**
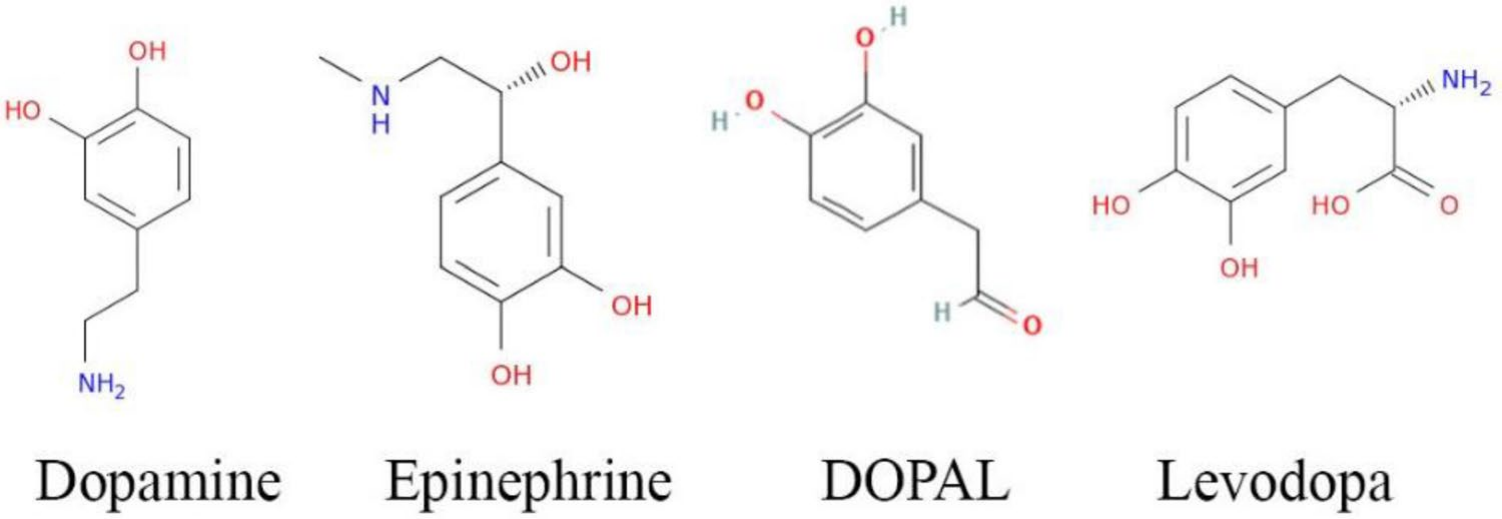
The chemical structure of catechols.

In the present study, starting from comparing the modulating effects of dopamine and some of its metabolites, including epinephrine, 3,4-dihydroxyphenylacetaldehyde (DOPAL), and levodopa (Fig. 1), on the amyloid fibrillation of α-syn in micromolar concentrations, and followed by comparing and analyzing the effects of metabolites on cytotoxicity of α-syn aggregates, we have investigated the interactions of α-syn aggregated species, produced in the presence of catechols with brain mitochondria. Our data revealed that the assembly process of α-syn is significantly inhibited in the presence of all studied dopamine metabolites. Furthermore, tested catechols exhibited various levels of neuroprotective effects against apoptotic cell death induced by α-syn aggregates. Treatment with α-syn fibrillar aggregates led to significant permeabilization of mitochondrial membranes, increase in mitochondrial reactive oxygen species (ROS), loss of membrane potential and mitochondrial swelling. Relating to the species produced in the presence of catechols, however, mitochondrial damage was significantly diminished, suggesting that these structures have lost their ability to cause mitochondrial membrane impairment. We believe that our data may provide a connection between different degrees of modifications induced by catechols influencing the membrane-binding-dependent physiological roles of α-syn.

## Results and discussion

### Catechols redirect the assembly process of α-syn toward the formation of oligomeric structures

**Figure 2:**
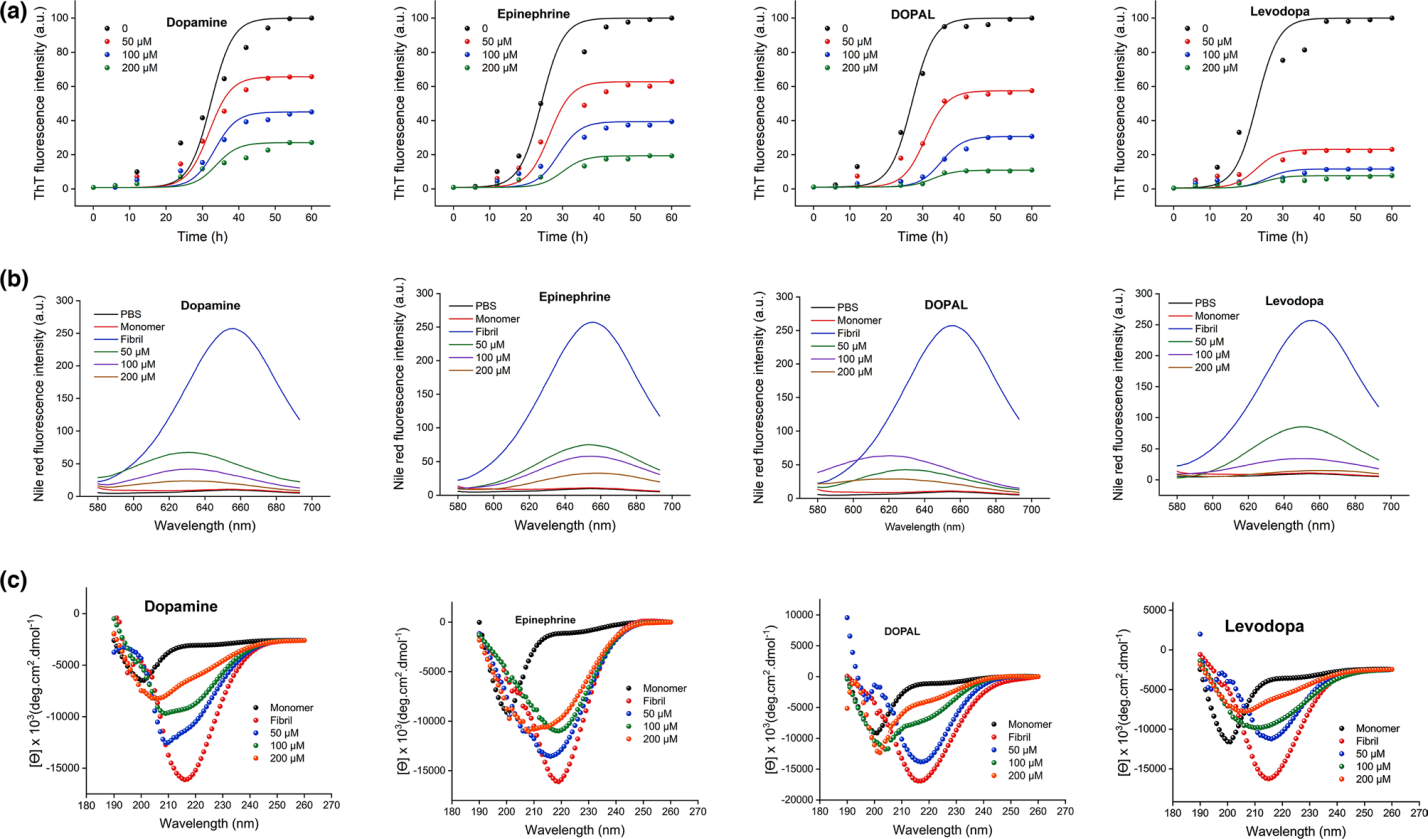
The effect of catechols on the α-syn amyloid fibril formation. Protein samples (200 µM) were incubated at 37 °C either alone or with increasing concentrations of catechols for 60 h. (**A**) The kinetics of α-syn amyloid fibrillation indicated by increasing fluorescence intensity of ThT at 485 nm. (**B**) Changes in the surface hydrophobicity of α-syn as monitored by NR fluorescence assay. (**C**) Far-UV CD spectra of α-syn incubated alone or in the presence of increasing concentrations of catechols. Data are expressed as mean ± SD with n = 5.

The kinetics of α-syn aggregation (200 µM) upon incubation alone or with increasing concentrations of catechols (50, 100, and 200 µM) was monitored by the ThT assay. The control experiments excluded the possibility of any interference of these catechols at studied concentrations with ThT and NR fluorescence assays (Supplementary Fig. 1). For protein samples incubated alone, the ThT fluorescence pattern followed sigmoidal kinetics (Fig. 2A), which is in agreement with the nucleation-dependent polymerization model of α-syn^20^. The addition of metabolites was found to inhibit the amyloidogenic assembly of α-syn in a concentration-dependent manner with a substantial inhibition at higher concentrations (Fig. 2A and Supplementary Fig. S2). The obtained results confirm previous reports, indicating inhibition of α-syn fibrillation by dopamine and other catechols^12,13,21–30^. While all tested metabolites were able to prevent amyloid fibrilization of α-syn, levodopa was found to be the most effective (Fig. 2A and Supplementary Fig. S2). To evaluate the ability of catechols in preventing the secondary nucleation of α-syn fibrillization, the seeding experiments were also carried out by adding sonicated preformed α-syn fibrils (5% v/v) to the ThT fluorescence assay. As shown in Supplementary Fig. S3, the ability of all catechols except DOPAL to inhibit amyloid fibril formation of α-syn was significantly decreased, indicating the inability of dopamine, epinephrine, and levodopa, in suppressing the secondary pathways in the course of the fibrillation process^31,32^. The conformational changes of incubated samples during the assembly process were probed by NR. As shown in Fig. 2B, for samples incubated in the absence of metabolites, a significant enhancement in NR fluorescence intensity was observed, indicative of solvent-exposure of hydrophobic regions of the protein. However, a considerable dose-dependent decrease in NR fluorescence intensity was observed in samples treated with metabolites (Fig. 2B and Supplementary Fig. S4). This observation indicates that the formation of partially folded intermediates with enhanced surface-exposed hydrophobic clusters, as critical species for amyloid fibril formation of α-syn^33^, has been prevented in the presence of catechols, in accordance with an earlier report^26^. This may be explained by the ability of dopamine and its metabolites to bind α-syn through nonspecific hydrophobic interactions^23,24^ mediated by the aromatic ring of catechols and the hydrophobic side chains of protein^27^.

**Figure 3:**
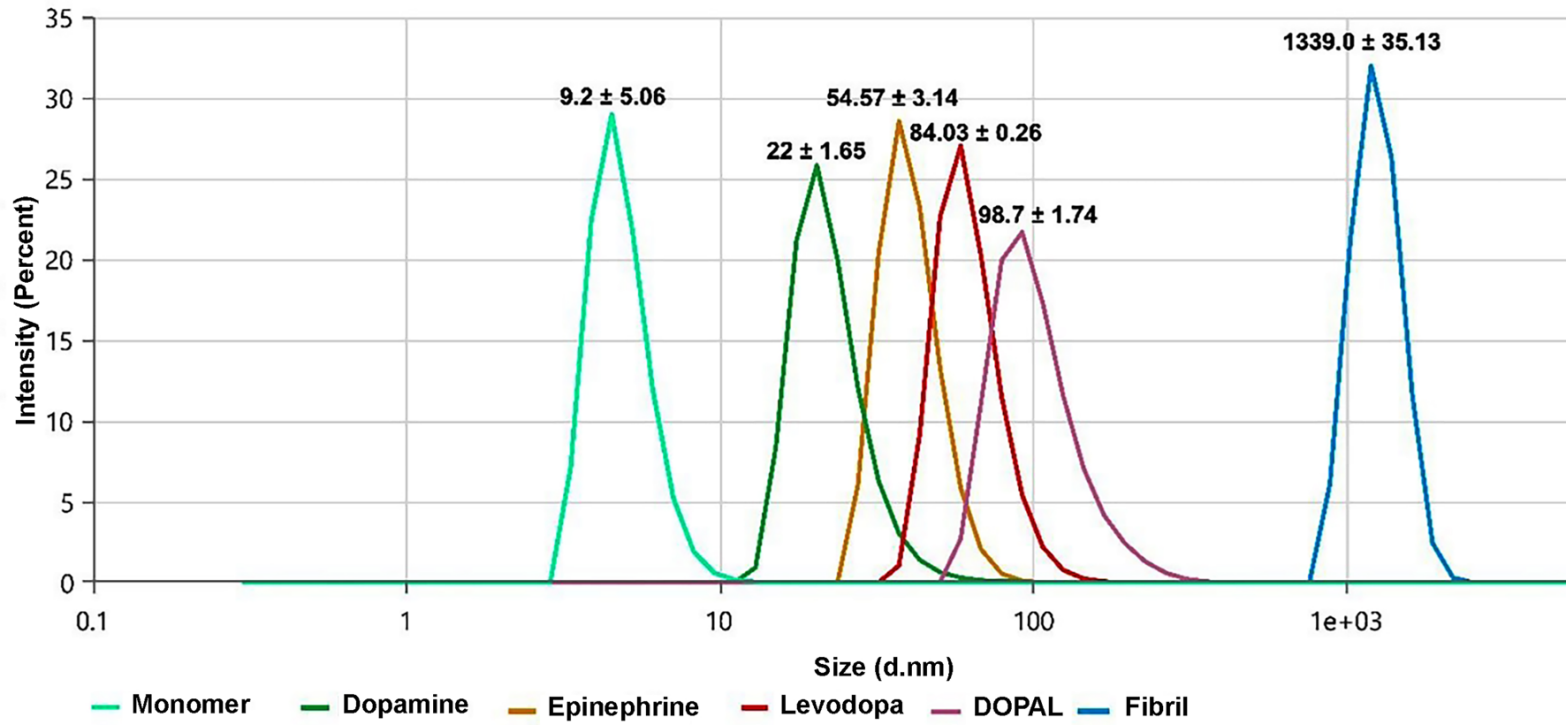
Size distribution of α-syn aggregates incubated alone (blue line) or with 200 µM dopamine (green line), epinephrine (yellow line), DOPAL (purple line), or levodopa (red line). Size distribution of monomer is also indicated (light green line). Results clearly indicate significant inhibition of α-syn assembly process mediated by catechols. All measurements were performed at pH 7.4 and room temperature (25 °C). Data are expressed as mean ± SD with n = 5.

Next, far-UV CD spectroscopy was applied to probe the secondary structural changes of α-syn in the absence and presence of metabolites. During incubation in amyloidogenic conditions, far-UV CD spectrum of α-syn was changed from a predominantly unordered structure of native protein to a well-defined β-structure, characterized by a negative peak at 218 nm, confirming the formation of amyloid fibrils (Fig. 2C). The CD spectra of all samples containing various concentrations of metabolites exhibited a dose-dependent inhibition of α-syn fibrillization by undergoing a significant decrease in β-sheet content along with an increase in unordered random coil structures (Fig. 2C and Supplementary Fig. S5), confirming earlier reports^26,29^. The capacity of metabolites to diminish the amount of β-sheet structures was as follows: levodopa > epinephrine > dopamine > DOPAL (Supplementary Fig. S5), suggesting the highest efficiency of levodopa in modulating the assembly process of α-syn, which is consistent with ThT fluorescence data (Fig. 2A). In the next step, DLS was used to determine size distribution of protein species produced in the absence and presence of 200 µM catechols. As shown in Fig. 3, for the protein samples incubated alone, the size distribution pattern shifted to very large-sized species (with an average diameter of 1339.0 ± 35.13) indicating the formation of amyloid fibrils. For samples incubated with metabolites, however, size distribution of aggregates shifted to smaller-sized species, suggesting that assembly process of α-syn has been inhibited, in accordance with fluorescence assays and CD measurements (Fig. 2).

**Figure 4:**
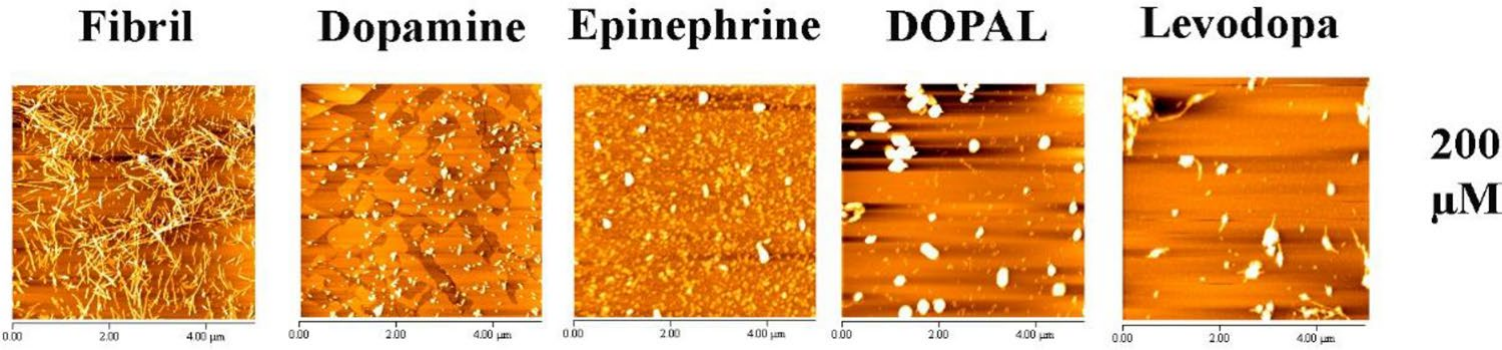
AFM images of protein samples incubated alone or in the presence of catechols (200 µM), for 60 h.

Finally, to observe the morphology of produced structures directly, the α-syn species formed at the end of the fibrillation process were visualized by atomic force and fluorescence microscopes. As illustrated in Fig. 4 and Supplementary Fig. S6, images recorded after 3 days of incubation revealed the presence of well-defined and typical amyloid fibrils only for the samples incubated in the absence of metabolites. As can be clearly seen, at the highest concentration of catechols (200 µM), the formation of such amyloid fibrils was inhibited entirely and instead, heterogeneously sized, spherical aggregates were observed (Fig. 4 and Supplementary Fig. S6). These results are in agreement with published reports demonstrating that catechols and related compounds with vicinal hydroxy groups are effective inhibitors of α-syn amyloid fibrillation^21,26,34^ and further validate previous observations reported by several studies^12,13,22–26,29,30,35^, indicating the appearance of off-pathway globular species as dominant species in the presence of dopamine or its metabolites. We suggest that inhibition of α-syn amyloid fibril formation is related to the catechols-induced covalent/non-covalent modifications reported previously^12,13,16,21–25,28,36^, affecting both cytotoxic assembly and membrane-binding properties of the protein.

### Catechols-induced globular species of α-syn exhibit reduced cytotoxicity

**Figure 5:**
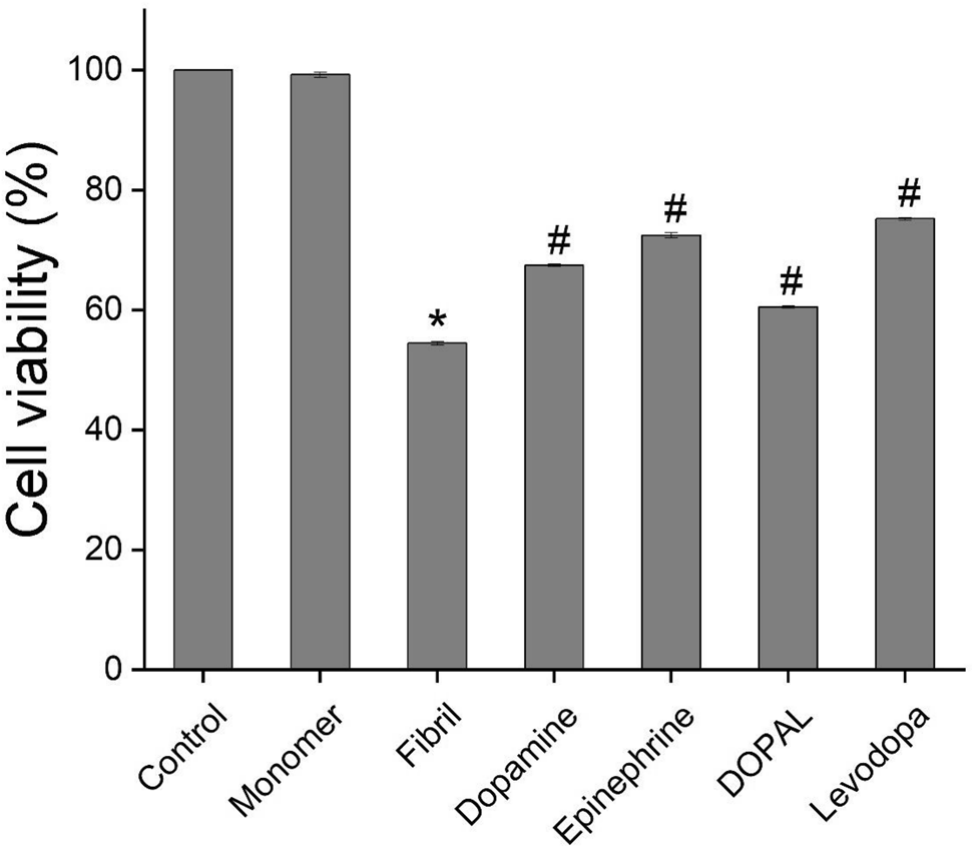
Cytotoxicity evaluation of α-syn aggregates (20 µM) produced in the absence or presence of 200 µM catechols. Further details relating to the experimental procedure are provided in the Materials and methods section. Data are expressed as mean ± SD with n = 5. ^*^p < 0.001, significantly different from control cells. ^#^p < 0.001, significantly different from cells exposed only to α-syn amyloid fibrils.

**Figure 6:**
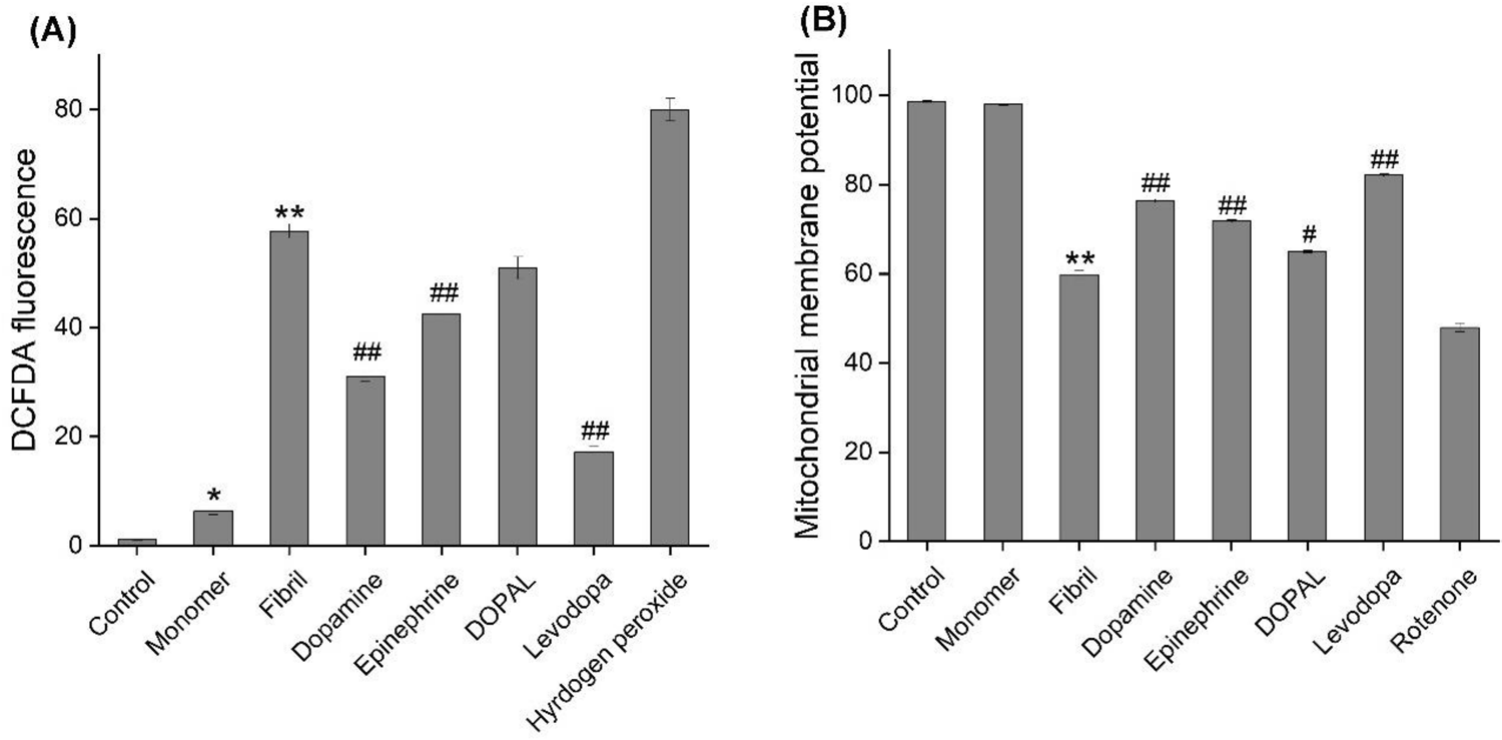
The effect of catechols on the α-syn aggregates-induced intracellular ROS and mitochondrial membrane potential in SH-SY5Y cell evaluated by flow cytometry. (**A**) and (**B**) Plots of the fluorescence intensities of DCFDA and Rhodamine 123 probes in cells exposed to 20 µM α-syn aggregates, produced in the absence or presence of 200 µM catechols, respectively. The effect of hydrogen peroxide and rotenone on intracellular ROS content and mitochondrial membrane potential, respectively, are also indicated. ^*^p < 0.05, ^**^p < 0.01 significantly different from control cells. Data are expressed as mean ± SD with n = 3. ^#^p < 0.05, ^##^p < 0.01 significantly different from cells exposed only to α-syn amyloid fibrils.

The cytotoxicity of α-syn aggregates produced in the absence or presence of catechols was evaluated using MTT-based cell viability test, intracellular ROS and mitochondrial membrane potential measurements, and Annexin V/PI double staining cell death assay. As reported recently^37^, treatment of SH-SY5Y cells with amyloid fibrils of α-syn led to a dose- and time-dependent decrease in viability of cells (Supplementary Fig. S7). In case of incubated samples containing increasing concentrations of metabolites, a dose-dependent decrease in toxicity was observed (Fig. 5 and Supplementary Fig. S8), which could be due to a substantial decrease in solvent-exposed surface regions of the protein as depicted in Fig. 2B. Although none of the metabolites, even at 200 µM, was able to prevent completely cell death induced by 20 µM α-syn fibrils (Fig. 5), levodopa exhibited the highest efficiency, what is coherent with its efficiency in modulation of α-syn fibrillation (Figs. 2 and 3). The cytotoxicity of aggregates produced alone or in the presence of metabolites was further examined by measuring intracellular ROS content and mitochondrial membrane potential, since two biochemical parameters significantly altered upon exposure of cells to α-syn aggregates^18,38^. Based on the flow cytometric results presented in Fig. 6 and Supplementary Fig. S9, exposure of SH-SY5Y cells to 20 µM α-syn fibrils resulted in a significant enhancement in intracellular ROS content and a considerable loss of mitochondrial membrane potential evaluated by DCFDA and rhodamine 123 fluorophores, respectively. These results indicate that α-syn-induced mitochondrial dysfunction characterized by increased levels of ROS and mitochondrial membrane depolarization, in agreement with previous reports^18,37,38^. The excess production of ROS may be attributed to the α-syn-induced deficiency in mitochondrial electron transfer chain complexes and especially complex I^39^, which in turn, can exacerbate mitochondrial impairment and promote oxidative damage to macromolecules leading to activation of cell death pathways, including mitochondria-mediated apoptosis^40^.

**Figure 7:**
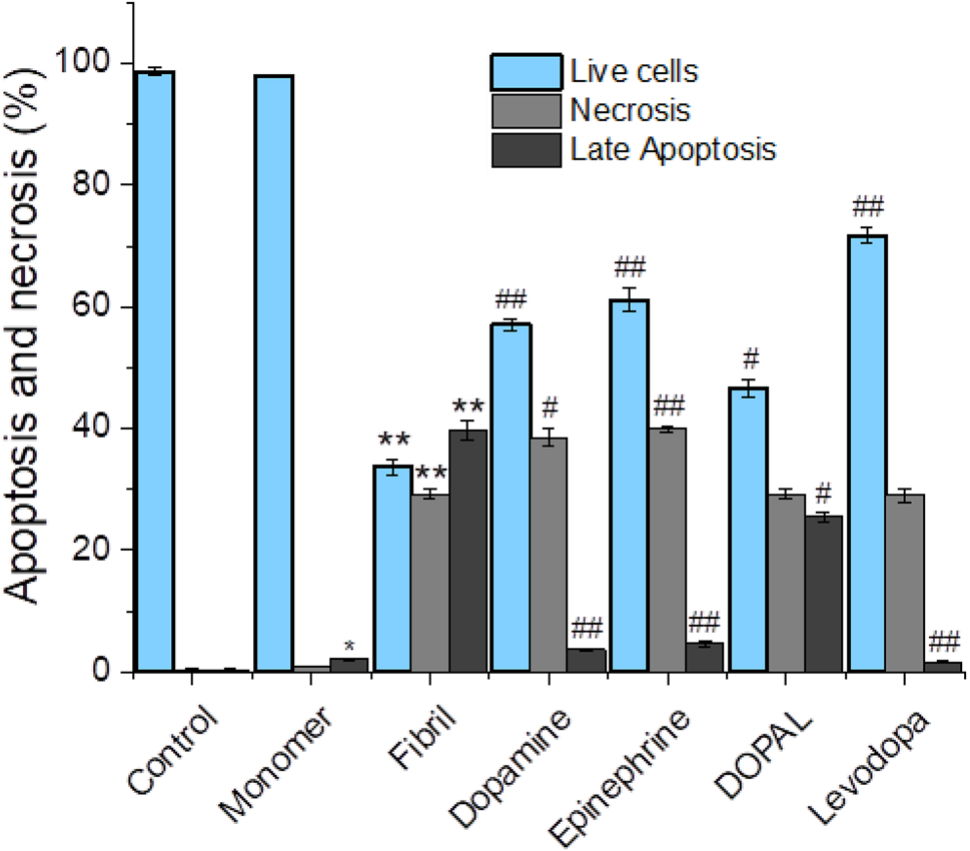
The effect of catechols on the α-syn aggregates-induced cell death in SH-SY5Y cell evaluated by flow cytometry. The percentages of live cells, late apoptotic and necrotic cells after 24 h post-treatment. All tested catechols, except DOPAL, were able to attenuate significantly toxicity related to α-syn amyloid fibrils via decreasing the α-syn amyloid fibril-induced apoptotic cell death. Data are expressed as mean ± SD with n = 3. ^*^p < 0.05, ^**^p < 0.01 significantly different from control cells. ^#^p < 0.05, ^##^p < 0.01 significantly different from cells exposed only to α-syn amyloid fibrils.

These cytotoxic effects of α-syn aggregates were attenuated considerably in the presence of metabolites, except DOPAL, with greater efficiency compared to levodopa (Fig. 6 and Supplementary Fig. S9). These cytoprotective properties of metabolites were verified further by investigating their capacity to prevent α-syn fibrils-induced cell death evaluated by Annexin V/PI double staining assay that is designed for detection of both apoptotic and necrotic cell deaths. The results presented in Fig. 7 and Supplementary Fig. S10, indicate clearly the involvement of both apoptosis and necrosis pathways in cell death induced by 20 µM α-syn aggregates. While neuronal cell death through both apoptosis and necrosis pathways has been reported by several amyloidogenic proteins^41^, there have not yet been reported any studies indicating necrotic cell death induced by α-syn^42^, and data obtained through in vitro studies indicate apoptosis as the primary mechanism^43–45^.

A possible explanation for our observation might, in part, be related to the methodology used to evaluate cell death. In most studies reporting α-syn-induced apoptotic cell death, authors have treated cells with very low concentrations (ranging from 0.05 nM to 10 µM) of α-syn^43–45^. Interestingly, Geci et al.^46^ indicated that depending on concentration, Aβ_25–35_ can induce apoptosis at low concentrations and necrosis at high concentrations. Accordingly, we suggest that at a high concentration (20 µM) amyloid fibrils of α-syn may activate pathways involved in necrosis, which is an unordered and accidental mode of cell death. Our observations confirm those reported by Okoshi et al.^47^ indicating that β2-microglobulin fibrils induce cell death through both apoptotic and necrotic pathways. An alternative explanation may be related to the presence of α-syn fibrillation products (including prefibrillar oligomers and amyloid fibrils), which are known to induce divergent cellular death pathways^48,49^ in incubated solutions used for the cell death assay.

As shown in Fig. 7 and Supplementary Fig. S10, the cell death induced by α-syn aggregates was considerably attenuated in the presence of metabolites, where DOPAL and levodopa exhibited the lowest and highest efficiency, respectively. The lower efficiency of DOPAL in reducing cytotoxicity related to α-syn fibrils may be related to the toxic nature of DOPAL-modified α-syn species^16,17^. According to the results, an increase in the population of live cells is accompanied by a decrease in α-syn amyloid fibril-induced apoptotic cell death (Fig. 7 and Supplementary Fig. S10). Based on this observation, we propose that the neuroprotection induced by catechols may be related to their ability to prevent the generation of toxic amyloid aggregates of α-syn, which are known to induce neuronal cell death through the mitochondria-mediated apoptotic pathway^42,50^. Further cellular experiments are needed to validate this proposition.

### Catechols modulate the interaction of α-syn aggregates with mitochondria

**Figure 8:**
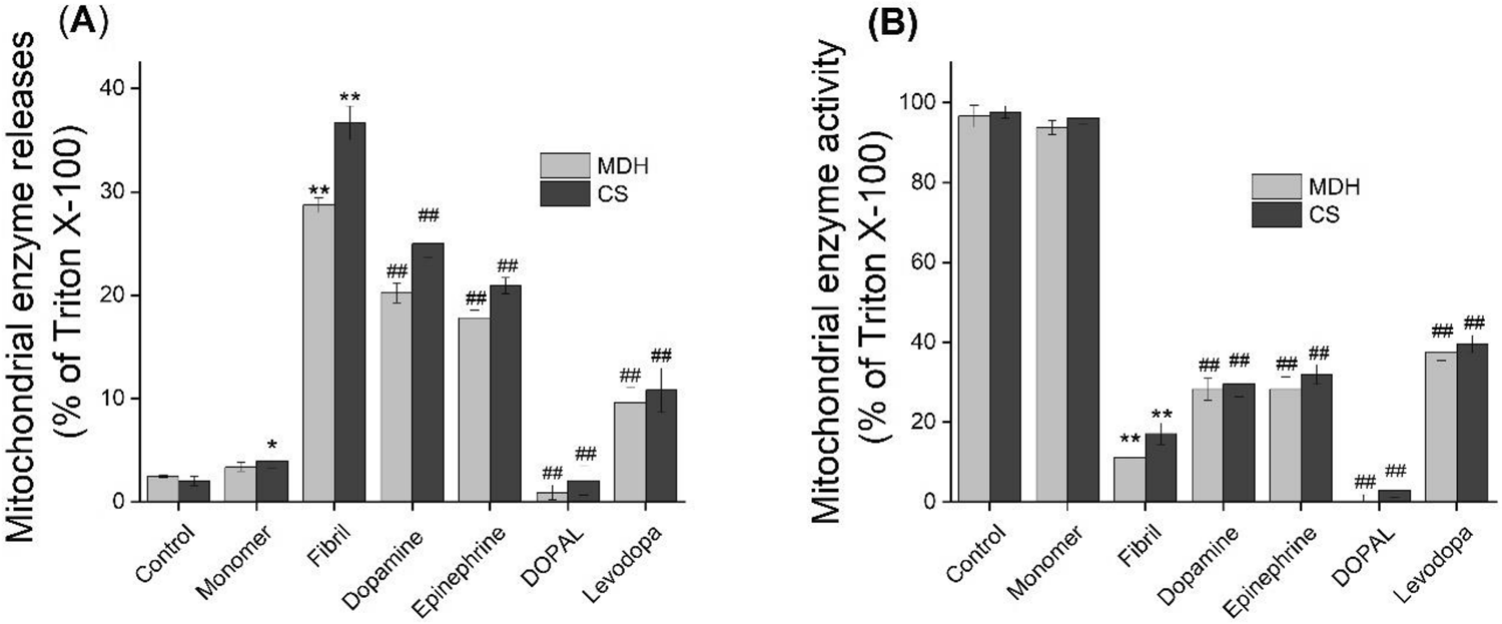
The effect of catechols on the α-syn aggregates-induced mitochondrial enzyme release (**A**) and enzyme activity inhibition (**B**). Mitochondrial suspensions (1 mg/mL) were incubated alone or with 20 µM α-syn aggregates, produced in the absence or presence of 200 µM catechols. Data are expressed as mean ± SD with n = 3. ^*^p < 0.05, ^**^p < 0.01, significantly different from control. ^##^p < 0.01 significantly different from mitochondria exposed only to α-syn amyloid fibrils.

The results obtained by extensive studies indicate the binding and accumulation of amyloidogenic peptides and proteins including α-syn on mitochondria, suggesting mitochondrial membrane as a potential target of toxicity. Therefore, in the second part of the study, the effect of catechols on the interaction of α-syn aggregates with lipid bilayer was investigated using the brain mitochondria as an in vitro biological model. This was achieved using a range of assays, including mitochondrial membrane permeabilization and enzyme activity determination, mitochondrial ROS content assay, mitochondrial membrane potential measurement, and mitochondrial swelling analysis. The permeabilization of mitochondrial membranes was investigated following an approach involving the release of mitochondrial MDH and CS, as described previously^51^. Exposure to α-syn amyloid fibrils of mitochondrial suspension resulted in the release of both mitochondrial enzymes in a concentration-dependent manner (Supplementary Fig. S11A). Similarly, a dose-dependent inhibition of MDH and CS activity was observed when we added α-syn aggregates to mitochondrial supernatant containing total enzymes released by Triton X-100 (Supplementary Fig. S11B). As depicted in Fig. 8, the ability of α-syn aggregates, produced in the presence of metabolites, to permeabilize mitochondrial membrane as well as inhibit MDH and CS activities was significantly decreased, where those incubated with levodopa exhibited the most efficiency. Interestingly, for samples containing DOPAL, detectable enzyme activities were not observed neither relating to membrane permeabilizing assays nor mitochondrial enzyme activity measurements (Fig. 8). A plausible explanation for this observation might be related to the capacity of DOPAL to modify proteins^52,53^, leading to inhibition of enzyme activity^52–54^.

**Figure 9:**
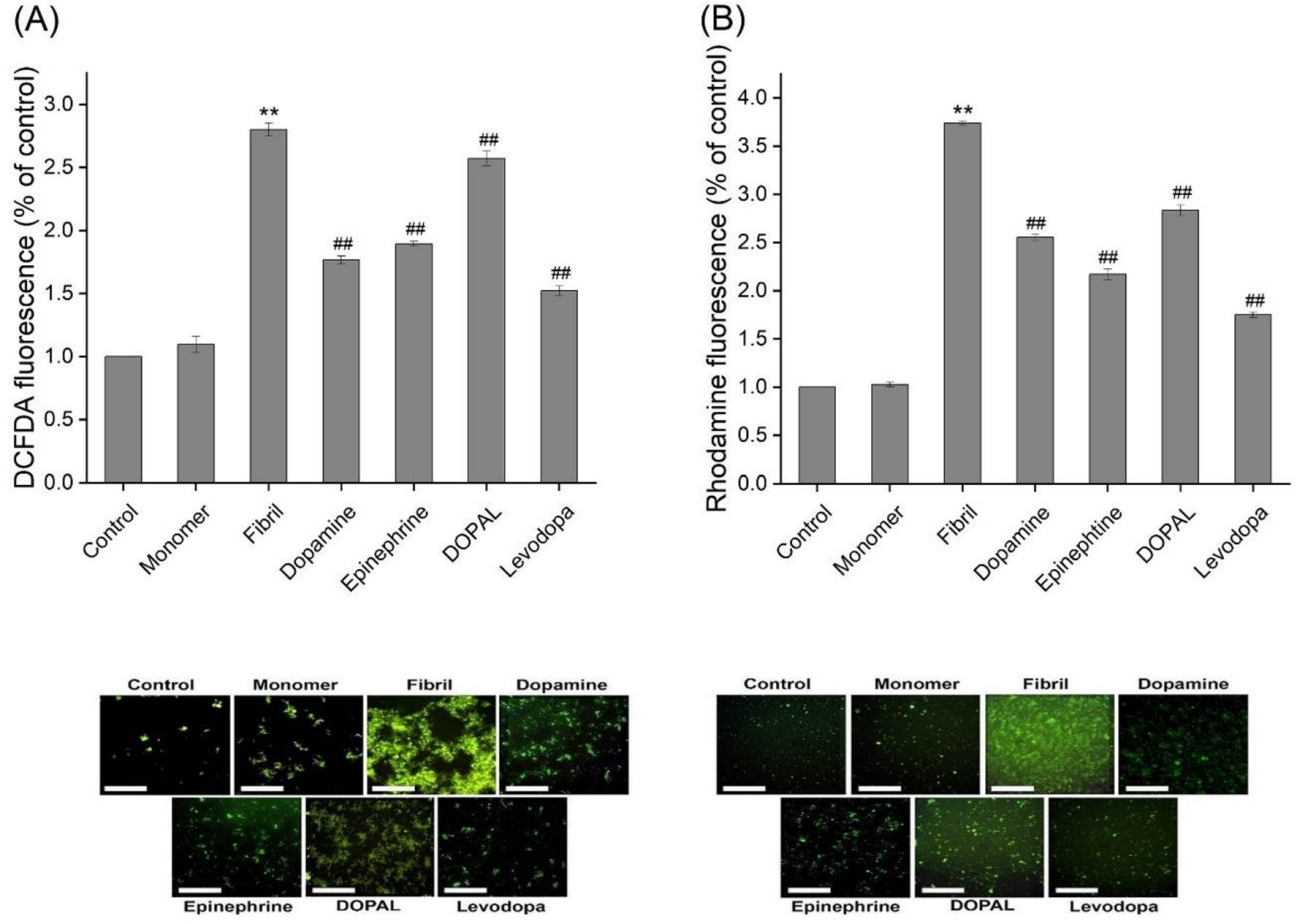
The effect of catechols on the α-syn aggregates-induced mitochondrial ROS enhancement (**A**) membrane depolarization (**B**). Mitochondrial suspensions (1 mg/mL) were incubated alone or with 5 µM α-syn aggregates, produced in the absence or presence of 200 µM catechols. Untreated mitochondria (control) and those treated with 5 μM α-syn monomer are also indicated. Low panels show fluorescence microscopy images of incubated samples. The scale bar represents 100 μm. Data are expressed as mean ± SD with n = 3. ^**^p < 0.01 significantly different from control. ^##^p < 0.01 significantly different from mitochondria exposed only to α-syn amyloid fibrils.

Since the integrity of mitochondrial membranes is necessary for biochemical assays relating to mitochondrial functionality, 5 µM α-syn aggregates were used for subsequent experiments, in which slight, if any, membrane permeabilization was observed (Supplementary Fig. S11A). The data presented in Fig. 9A, show the effect of α-syn aggregates produced alone or in the presence of catechols on the ROS content of brain mitochondria investigated by DCFDA fluorescence measurement and microscopic imaging. According to the obtained data, it can be concluded that exposure to α-syn amyloid fibrils results in a remarkable enhancement in mitochondrial ROS content. This observation may be attributed to the high capacity of α-syn for interaction with and binding to biological membranes which have negatively charged surfaces, such as mitochondria^39,55^. The obtained results confirm previously published reports^50,56^, indicating preferential binding of α-syn aggregates to mitochondria leading to increased oxidative stress and mitochondrial impairment. However, the aggregated species produced in the presence of 200 µM metabolites, except DOPAL, exhibited decreased efficacy in causing ROS enhancement (Fig. 9A). Similarly, we observed a significant decrease in the capacity of α-syn aggregates (5 µM) incubated in the presence of 200 µM metabolites to perturb mitochondrial membrane potential (Fig. 9B). According to these data, it can be concluded that the capacity of α-syn aggregates to increase mitochondrial ROS content and depolarize mitochondrial membrane^37^ has been substantially inhibited in the presence of catechols.

**Figure 10:**
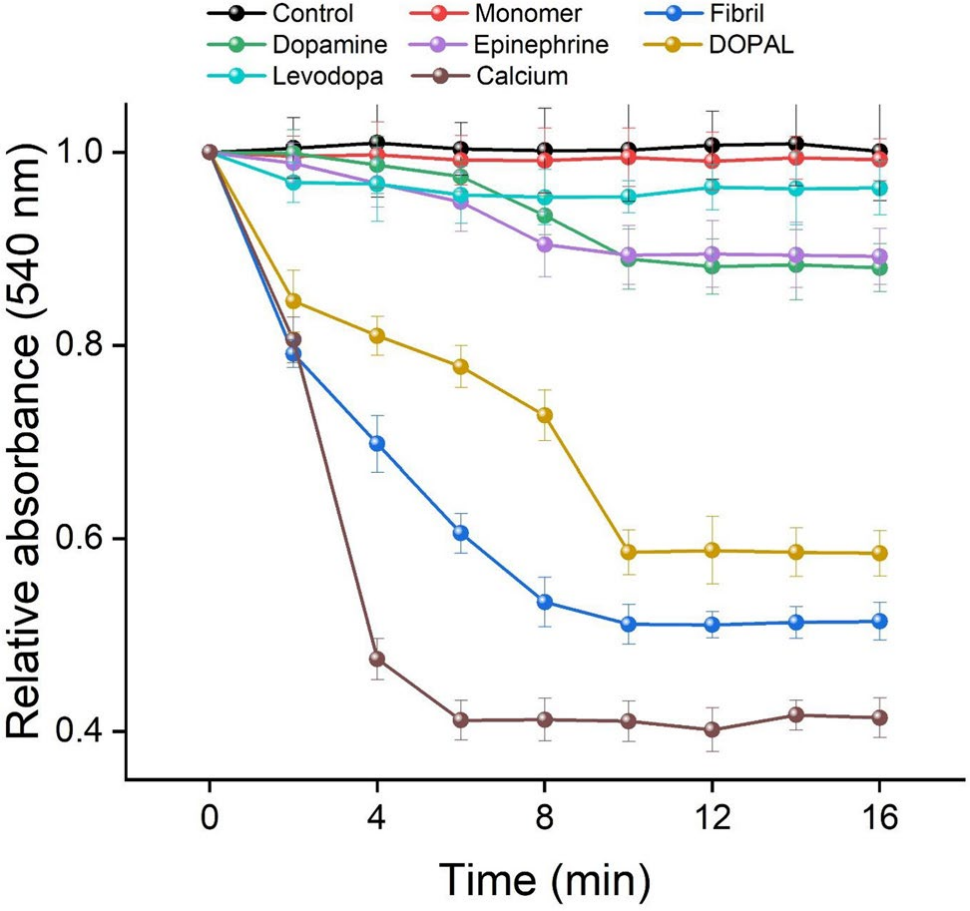
The effect of catechols on the swelling kinetics of brain mitochondria induced by α-syn aggregates. Mitochondrial suspensions (1 mg/mL) were incubated alone or with 5 µM α-syn aggregates, produced in the absence or presence of 200 µM catechols. Untreated mitochondria (control) and those treated with 5 μM α-syn monomer or 2 mM calcium are also indicated. Data are expressed as mean ± SD with n = 3.

It has been known that influx of ions/metabolites with molecular weight < 1.5 KDa upon membrane depolarization can result in mitochondrial swelling characterized by a decrease in absorbance. Figure 10 shows swelling of the brain mitochondria treated with 5 μM α-syn aggregates produced alone or in the presence of increasing concentrations of metabolites. While α-syn fibrils were very effective in causing mitochondrial swelling, no significant decrease in absorbance (540 nm) was observed in mitochondria treated with the aggregates produced in the presence of these metabolites (Fig. 10). However, the exposure of mitochondria to DOPAL-modified α-syn species caused a considerable reduction in absorbance (Fig. 10). Based on the obtained data, we suggest that globular species produced in the presence of metabolites have lost their ability to damage mitochondria. These results are in agreement with other published reports indicating reduced membrane-binding properties of α-syn upon exposure to dopamine^57^ and DOPAL^28^.

Although the physiological functions of α-syn remain poorly understood at presynaptic terminals, it is thought that many of these protein functions are attributed to its membrane binding properties. This feature generally mediates via its N-terminal lipid binding domain containing 11-mer repeats centered on a KTKEGV consensus sequence^58^ and a cryptic mitochondrial targeting signal^39^, suggesting that modification of these sequences can modulate the membrane-binding features of the protein. In particular, lysine residues within the N-terminal repeats play a crucial role in the interaction of protein with membranes^59^. On the basis of these results, we propose that decreased mitochondrial binding capacity of α-syn may be related to catechols-induced covalent adducts with lysine residues^16,21,28^ or non-covalent hydrophobic interactions of catechols with N- and C-terminal regions of α-syn^23,24,27,36^ affecting both cytotoxic assembly process and membrane-binding properties of the protein.

## Conclusions

In the present study, we show the capacity of dopamine and some of its metabolites to modulate the cytotoxic amyloid fibrillation and mitochondrial-binding properties of α-syn. The obtained results indicate that the catechols-induced species showed decreased cytotoxicity, which correlates with a reduction of surface-exposed hydrophobic patches, and significantly lost their ability to bind and cause mitochondrial impairment. The difference in the capacity of metabolites in modulating cytotoxic aggregation and membrane binding properties of α-syn may be related to the nature of interactions occurring between catechols and protein. While some in vitro and in vivo studies suggest that formation of such soluble oligomers induced by catechols leads to toxicity^17,21,60,61^, other reports indicate that catechols-induced globular species are non-toxic^25,26,62,63^, which is in accordance with our results. These findings may suggest that depending on methodology and experimental conditions, the structural properties of oligomeric species may differ. For instance, while Follmer et al.^28^ showed that DOPAL-induced modification of α-syn reduced the binding of protein to synaptic-like vesicles, Plotegher et al.^17^ reported permeabilization of synaptic vesicles promoted by DOPAL-induced oligomers of α-syn. In another study, Zhou et al.^36^ found that DOPAC at low concentration binds α-syn non-covalently and promotes the formation of transient oligomers, but covalent modifications of protein leading to the formation of stable oligomeric species occur at high concentrations. This observation may propose a catechol concentration-dependent modification of α-syn, which can lead to the production of oligomers with different conformational and, consequently toxicity features. Based on these data, we suggest that low molar ratios of catechol/protein used in this study may promote non-covalent interactions between catechols and α-syn leading to the formation of non-toxic oligomers stabilized by hydrophobic interactions. Further studies are needed to confirm this hypothesis. In accordance with this proposition, α-syn fibrillation inhibition through the catechols-induced formation of α-syn oligomers promoted by conformational changes rather than covalent modifications has been reported as a novel mechanism by which catechols, including dopamine metabolites, can modulate α-syn fibrilization^23,24,27^. In summary, the obtained data indicate neuroprotective effects of catechols against cytotoxicity and mitochondrial damage induced by α-syn amyloid fibrils, suggesting that dyshomeostasis of intracellular levels of catechols may participate in the development of PD through promoting the formation of insoluble toxic aggregates.

## Materials and methods

### Materials

Wild-type (WT) α-syn plasmid (pT7-7-WT), which encoded the 140 amino acids of protein, was obtained from Addgene. Isopropyl β-d-1-thiogalactopyranoside (IPTG) was purchased from CinnaGen, Iran. Thioflavin T (ThT), Nile red (NR), dihydrodichlorocarboxyfluorescein diacetate (DCFDA), rhodamine 123, 3-(4,5-dimethylthiazol-2-yl)-2,5diphenyltetrazolium bromide (MTT), dopamine, epinephrine, levodopa, cell culture medium Dulbecco modified Eagle medium (DMEM), and penicillin–streptomycin antibiotics were purchased from Sigma (St. Louis, MO, USA). DOPAL was purchased from Cayman Chemicals. Fetal bovine serum was purchased from Gemini Bio (Woodland, CA). All other chemicals were obtained from Merck (Darmstadt, Germany) and were reagent grade.

### Expression and purification of α-synuclein

The expression of untagged recombinant WT α-syn was performed in *E. coli* BL21 (DE3) in a pT7-7-based expression system according to Hoyer et al.^64^ with some modifications. Briefly, BL21-competent cells were grown in LB (overnight 16–19 h) in the presence of ampicillin (100 μg /mL). After inducing with 1 mM IPTG and running for 4 h at 37 °C, cell pellets were harvested by centrifugation at 6000 rpm for 20 min at 4 °C. The cell pellet was resuspended in 20 mM Tris–HCl (pH 8.0) containing 1 mM EDTA and 1 mM PMSF and lysed by multiple sonication cycles followed by boiling for 15 min. Next, the suspension was centrifuged at 18,000*g* for 20 min at 4 °C. The supernatant was collected, and ammonium sulfate was slowly added (0.36 g/mL) to precipitate the α-syn protein selectively. The solution was stirred for 1 h at 4 °C and centrifuged at 18,000*g* for 20 min at 4 °C. The pellet was dissolved in 20 mM Tris–HCl (pH 8), sterile-filtered through a 0.22 µm syringe filter, and loaded onto a 5-ml HiTrap Q FF anion exchange chromatography column (GE Healthcare). α-syn was eluted at 300 mM NaCl and the pure protein (confirmed by SDS-PAGE electrophoresis)^65^ was dialyzed overnight against phosphate-buffered saline (PBS) (137 mM NaCl, 2.7 mM KCl, 10 mM Na_2_HPO_4_, 1.76 mM KH_2_PO_4_, pH 7.4). The protein concentration was determined by measuring the absorbance at 275 nm^64^. The purified protein was stored at – 75 °C in 1 mL aliquots.

### Sample preparation and α-synuclein amyloid fibril formation

Stock solutions of dopamine, epinephrine, and levodopa were prepared using deionized water as a solvent. The stock of DOPAL was formulated as a solution in methanol. The molar ratios of metabolite to protein used in this study were 0:1, 0.25:1, 0.5:1, and 1:1. The final concentration of methanol did not exceed 0.3% in the incubating solutions containing the highest concentration of DOPAL. For α-syn fibril formation, the protein was dissolved in PBS (pH 7.4) to a final concentration of 200 μM, and aliquots were incubated in the absence or presence of various concentrations of metabolites at 37 °C under constant stirring at 1,000 rpm for 72 h.

### α-syn amyloid fibrillation and characterization

All fluorescence experiments were carried out on a Cary Eclipse VARIAN fluorescence spectrophotometer. For monitoring the growth of amyloid fibrils, ThT fluorescence intensity of protein samples incubated without or with various concentrations of metabolites was determined using a mixture of 4 μM protein solutions and 10 μM ThT (prepared from 10 mM ThT stock solution in 25 mM sodium phosphate, pH 7.4, passed through a 0.22 μm filter paper) with fixed excitation at 440 nm and emission at 485 nm. Excitation and emission slit widths were set at 5 and 10 nm, respectively. To investigate the effect of catechols on the secondary nucleation of α-syn fibrillation process seeding experiment was conducted. Briefly, preformed amyloid fibrils of α-syn were centrifuged at 15,000×*g* for 10 min and re-suspended twice in PBS buffer to eliminate oligomers^66^. To prepare seeds, amyloid fibrils were sonicated for 3 min using a probe sonicator (5-s on, 5-s off) using 10% maximum power and 30% cycles^67^. The seeds (5% v/v) were added to protein solutions (200 µM) containing 20 µM ThT and 200 µM concentration of each catechols at a final volume of 200 µL^68^. The solutions were transferred in triplicates to a black 96-well clear bottom plate and sealed with Crystal Clear sealing tape followed by incubation at 37 °C while shaking at 1000 rpm. For NR fluorescence measurements, aliquots of protein solutions were removed at different time intervals and diluted to a final concentration of 2 μM in PBS (pH 7.4) containing 5 μM NR (prepared from 5 mM NR stock solution in methanol, passed through a 0.22 μm filter paper). Samples were excited at 530 nm, and emission spectra were recorded from 540 to 700 nm. For far-UV CD analysis, aliquots of incubated samples were removed and diluted to a final concentration of 33 μM. The spectra were recorded in the range of 190–260 nm using an AVIV 215 spectropolarimeter (Aviv Associates, Lakewood, NJ, USA) and a 1.00 mm path cell. The spectra were deconvoluted using the SELCON program on the online server DICHROWEB^69^, and the percent of α-helix, β-sheet, turns and unordered structures were determined for each sample. To investigate the size distribution of α-syn assemblies (200 µM) during fibril formation in the absence and presence of studied catechols (200 µM), dynamic light scattering (DLS) measurements was performed. Aliquots of protein and catechols (in a 1:1 molar ratio) were filtered through a 0.2 μm syringe filter followed by incubation under amyloidogenic conditions as mentioned above. Samples (at a final concentration of 66 μM) were illuminated using Zetasizer, Nano ZS instrument (Malvern Panalytical UK, Malvern, UK) at 25 °C, probing scattered light at 173° using a 630 nm light source. Measurements were performed in five repetitions.

Finally, the morphological characteristics of incubated samples were analyzed using atomic force and fluorescence microscopies. Briefly, 10 µL of samples (diluted to a final concentration of 2 µM with deionized water) were placed on freshly cleaved mica and dried at room temperature (RT). Images were acquired in non-contact mode using a quantitative AFM (Veeco-Auto Probe-PC Research). For fluorescence microscopy, 10 µL of incubated samples (diluted with PBS to a final concentration of 10 µM) were mixed with 10 µL of ThT solution (10 µM) and incubated for 10 min in a dark place at RT. The mixture was added on a clean glass slide and then air-dried. The images were captured on a fluorescence microscope (Axioskop 2 plus, Zeiss, Germany) at 20 × or 40 × magnifications.

### Cell toxicity assays

Human neuroblastoma cells (SH-SY5Y, NCBI code: C611, Pasteur Institute of Iran) were cultured in DMEM medium, supplemented with 10% fetal bovine serum, streptomycin (100 μg/mL) and penicillin (100 U/mL), and kept at 37 °C in a 5% CO_2_ humidified atmosphere. Cells were seeded in a 96-well plate at a density of 2 × 10^4^ cells/well (at a final volume of 100 µL/well), and incubated at 37 °C to adhere for 12 h. The medium was changed before incubation with α-syn amyloid aggregates. For cytotoxicity experiments, cells were treated with increasing concentrations (0–20 μM) of α-syn fibrils and incubated for 24 h and 48 h. To evaluate the involvement of anti-amyloidogenic activity of metabolites against toxicity induced by α-syn amyloid fibrils, aliquots of protein samples aged without or with various concentrations of metabolites (0, 50, 100, and 200 μM) under amyloidogenic conditions were added to the cells (5, 10, and 20 μM) and left for 24 h. Cells treated with PBS were used as a control. Cell viability was assessed using the conventional MTT reduction assay. Briefly, after treatment, the medium was replaced with 50 μL/well of MTT stock solutions (5 mg/mL in PBS), followed by incubation at 37 °C for 4 h. Solutions were aspirated, and cells were treated with 10 μL/well DMSO for 15 min, followed by absorbance reading at 570 nm on a microplate reader (BioTek). Results were expressed as a percentage of MTT reduction relative to the control cells, assuming that the absorbance of the control cells was 100%. The mitochondrial ROS content was measured using the oxidation-sensitive fluorescent probe DCFDA. The SH-SY5Y cells were cultured in 6-well dishes at 5 × 10^5^ cells/well density and incubated overnight. Then, cells were treated with 20 µM incubated samples aged alone or in the presence of 200 µM catechols and incubated for 6 h. Cells treated with 750 µM H_2_O_2_^70^ were used as a positive control. After incubation, 5 µM DCFDA was added to each Petri dish, and cells were incubated for an additional 30 min at 37 °C. After trypsinization and washing twice with PBS solution, the fluorescence was recorded with flow cytometry (Partec Cyflow, Germany) at the excitation wavelength of 488 nm. The quantification of ROS levels was analyzed using FlowJo Software (version 7.6, USA), and the obtained data were normalized to the control group. To evaluate changes in the mitochondrial membrane potential of SH-SY5Y cells, aliquots of incubated samples (20 μM) were added to cells, followed by incubation for 6 h. Cells treated with 10 µM rotenone were used as a positive control. After trypsinization and washing with PBS, cells were treated with 50 nM rhodamine 123 at 37 °C and supplied with 5% CO_2_ for 1 h. Then, SH-SY5Y cells were washed twice with DMEM culture medium and resuspended in 1 mL PBS. Finally, the percentages of rhodamine 123-positive and rhodamine 123-negative SH-SY5Y cells were determined with flow cytometry (Paretc, Cyflow, Germany) using 488 nm and 525 nm as excitation and emission wavelengths, respectively. For the cell death assay, 5 × 10^5^ cells/well were seeded in a 12-well plate and incubated for 24 h, followed by treatment with 20 μM of incubated samples. After treatment, cells were detached by trypsin and pelleted through centrifugation at 250 g for 5 min at 4 °C, followed by resuspension in PBS. The SH-SY5Y cells were stained with the Annexin V-FITC/PI double staining apoptosis detection kit (BioLegend, 640914) at RT for 20 min, according to the manufacturer datasheet. Subsequently, the samples were analyzed by flow cytometry (Partec, Cyflow, Germany). For each experiment, 15,000 events per sample were read. The different quadrants Q1, Q2, Q3, and Q4 indicate necrosis, late apoptosis, early apoptosis, and live cells, respectively.

### Mitochondrial toxicity assays

#### Preparation of rat brain mitochondria

All animal experiments were performed in accordance with the Institutional Animal Care and Use Committee (IACUC), Tehran University of Medical Sciences. Maximal efforts were made to minimize suffering and detrimental effects to the rats by sharpening the guillotine blades and applying resolute and swift movements of the blade. All reagents for mitochondrial isolation were prepared according to Sims protocol^71^ and Zadali et al.^72^. Decapitation and brain removal of male rats was performed according to Sims protocol^71^. Briefly, male, albino, NMRI rats (150–200 g) were decapitated and brains were removed, washed, and then homogenized in 10 volumes of pre-cooled isolation buffer [10 mM Tris–HCl, 1 mM EDTA, and 0.32 M sucrose, 1% BSA (w/v) (pH 7.4)]. The homogenate was centrifuged at 3000*g* for 3 min, and the mitochondrial fraction from the resulting supernatant was centrifuged at 21,000*g* for 10 min. The new pellet was re-suspended in 15% (v/v) Percoll and centrifuged at 30,700*g* for 8 min. The material accumulated at the top of the gradient, which mostly contained myelin, was removed. The remainder suspension, containing both the synaptic and the non-synaptic mitochondria, was diluted with isolation buffer followed by centrifugation at 16,700*g* for 10 min. The supernatant was carefully removed and the bottom loose pellet was re-suspended in 10 mg/mL fatty-acid-free bovine serum albumin, and then centrifuged at 6900*g* for 10 min^63,64^. The supernatant was decanted and the mitochondrial pellet was collected and stored in liquid nitrogen for subsequent experiments. All centrifugation steps were carried out at 4 °C. Mitochondrial membrane integrity was confirmed by measuring the malate dehydrogenase (MDH) activity in isolated mitochondria before and after membrane disruption by Triton X-100^72^.

#### Treatment of mitochondria with incubated samples

Aliquots of solutions containing monomer or protein samples aged in the absence or presence of various concentrations (50, 100, 200 µM) of metabolites, or PBS as a control, were added to 200 μL of intact mitochondrial homogenates (1 mg/mL diluted with isolation buffer). The solutions were incubated for 30 min at 30 °C while gently stirred. For mitochondrial membrane permeabilization assay and mitochondrial enzyme activity measurement, we used various concentrations of incubated samples ranging from 0 to 20 µM. In the case of mitochondrial ROS content, membrane potential measurement, and mitochondrial swelling assay, incubated samples (at a final concentration of 5 µM) were applied, in which no significant membrane permeabilization was observed. The amyloid fibril concentrations are given in terms of the monomer protein concentration throughout the study. The incubated solutions were then used for the subsequent assays.

#### Mitochondrial membrane permeabilization assay

Mitochondrial membrane permeabilization was investigated following an approach involving the release of mitochondrial enzymes, as described previously^51^. Briefly, at the end of the incubation period, mitochondrial suspensions were centrifuged at 21,000*g* for 30 min, and the activity of two mitochondrial enzymes, including MDH and citrate synthase (CS), was determined in the supernatant using spectrophotometric assays described by Sottocasa et al.^73^ and Muller-Kraft and Babel^74^, respectively. Triton X-100 (at a final concentration of 0.5% (v/v)) was used as a positive control for maximum enzyme release. Data are expressed as a fraction of the maximum effect (Triton X-100): (measured signal − blank signal)/(maximum signal − blank signal)^51^. In some experiments, the membrane of intact mitochondria was completely disrupted by Triton X-100 (0.5% v/v). The mitochondrial suspension was then centrifuged, and the activity of MDH and CS in the supernatant was measured in the absence and presence of incubated samples (20 µM).

#### Measurement of ROS content and membrane potential of mitochondria

The ROS content and membrane potential of brain mitochondria were determined using fluorogenic probe DCFDA^75^, and mitochondrial membrane potential sensitive dye rhodamine 123^76^, respectively. Briefly incubated solutions were transferred into a nonbinding surface black with a dark bottom polystyrene 96-well plate (Corning Incorporated, 2 Alfred Road, Kennebunk ME 04,043 USA), followed by the addition of 1 μM DCFDA and 5 mM succinate for mitochondrial ROS measurement, or 20 nM rhodamine 123 for a membrane potential estimation. The plate was sealed with transparent sealing tape and loaded into a Synergy Hybrid Multi-Mode microplate reader (BioTek Instruments, Winooski, VT 05404-0998, USA). For ROS measurement, the fluorescence signal of DCFDA was recorded with excitation at 485 nm and emission at 530 nm. Mitochondrial membrane potential was estimated by measuring the fluorescence intensity of rhodamine 123 at excitation and emission wavelengths of 503 and 527 nm, respectively. All fluorescence measurements were done in triplicate at 37 °C for 2 h, and the mean of the three measurements was determined. During the measurements, the reaction medium containing mitochondria was continuously stirred. At the end of incubation, 10 μL of incubated samples were added on a clean glass slide and then air-dried. The images were captured on a fluorescence microscope (Axioskop 2 plus, Zeiss, Germany) at 20 X magnification.

#### Mitochondrial swelling assay

The mitochondrial homogenates were resuspended in 10 mM HEPES, 5 mM succinate, 250 mM sucrose, 1 mM ATP, 0.08 mM ADP, 2 mM K_2_HPO_4_, 1 mM DTT (pH 7.5), in a final concentration of 1 mg/mL of mitochondrial suspension. The swelling was initiated by adding monomers or aggregates produced in the absence or presence of metabolites. Calcium (2 mM) was used as a positive control for maximum swelling. Mitochondrial swelling was evaluated by following a decrease in the light scattering at 540 nm for 15 min at RT. For swollen mitochondria, light transmission is increased (i.e. a decrease in light scattering would indicate a dilution of mitochondrial solutes due to swelling)^37^. After subtracting the blank (buffer alone) from all values, mitochondrial swelling curves were graphed.

### Statistical analysis

All the results were average of at least three independent repeats and represented as mean ± standard error of the mean (SEM). The data were analyzed statistically using Microsoft Office Excel Version 2016. One-way analysis of variance (ANOVA) was used to compare the means for continuous variables. The p-values of 0.01 and 0.001 were considered as the level of statistical significance.

### Ethics statement

We confirm that all methods were performed in accordance with the relevant guidelines and regulations. Animal studies were performed after receiving approval from the ethics committee of Tehran University of Medical Science. All the ethical issues have been monitored during the project in accordance with the existing protocols. This study was carried out in accordance with ARRIVE guidelines (https://arriveguidelines.org).

## Supplementary Information

Supplementary Figures.

## Data Availability

The data that support the findings of this study are available from the corresponding author upon reasonable request.
