# Supplementary material for: Modulation of cytotoxic amyloid fibrillation and mitochondrial damage of α-synuclein by catechols mediated conformational changes
Source: Sci Rep. 2023 Mar 31;13:5275. doi: 10.1038/s41598-023-32075-9 (PMC10066314; doi:10.1038/s41598-023-32075-9)
Supplement: Supplementary file 1 — Supplementary Figures. [file 41598_2023_32075_MOESM1_ESM.docx]

**Supplementary information**

**Modulation of cytotoxic amyloid fibrillation and mitochondrial damage of α-synuclein by catechols mediated conformational changes**

Toktam Zohoorian-Abootorabi^1^, Ali Akbar Meratan^2,^*, Saeed Jafarkhani^3^, Vladimir Muronetz^4^, Thomas Haertlé^5^, Ali Akbar Saboury^1,^*

^1^Institute of Biochemistry and Biophysics, University of Tehran, Tehran 14176-14335, Iran

^2^Department of Biological Sciences, Institute for Advanced Studies in Basic Sciences (IASBS) 45137-66731, Zanjan, Iran

^3^ Division of Biomedical Engineering, Faculty of New Sciences and Technologies, University of Tehran, Tehran 57131-14399, Iran

^4^ Belozersky Institute of Physico-Chemical Biology, Lomonosov Moscow State University, Moscow 119991, Russia

^5^ National Institute of Agronomic and Environmental Research, 44316 Nantes, France

^⁎^***Corresponding Authors***: saboury@ut.ac.ir (A.A. Saboury) & a.meratan@iasbs.ac.ir (A.A. Meratan)


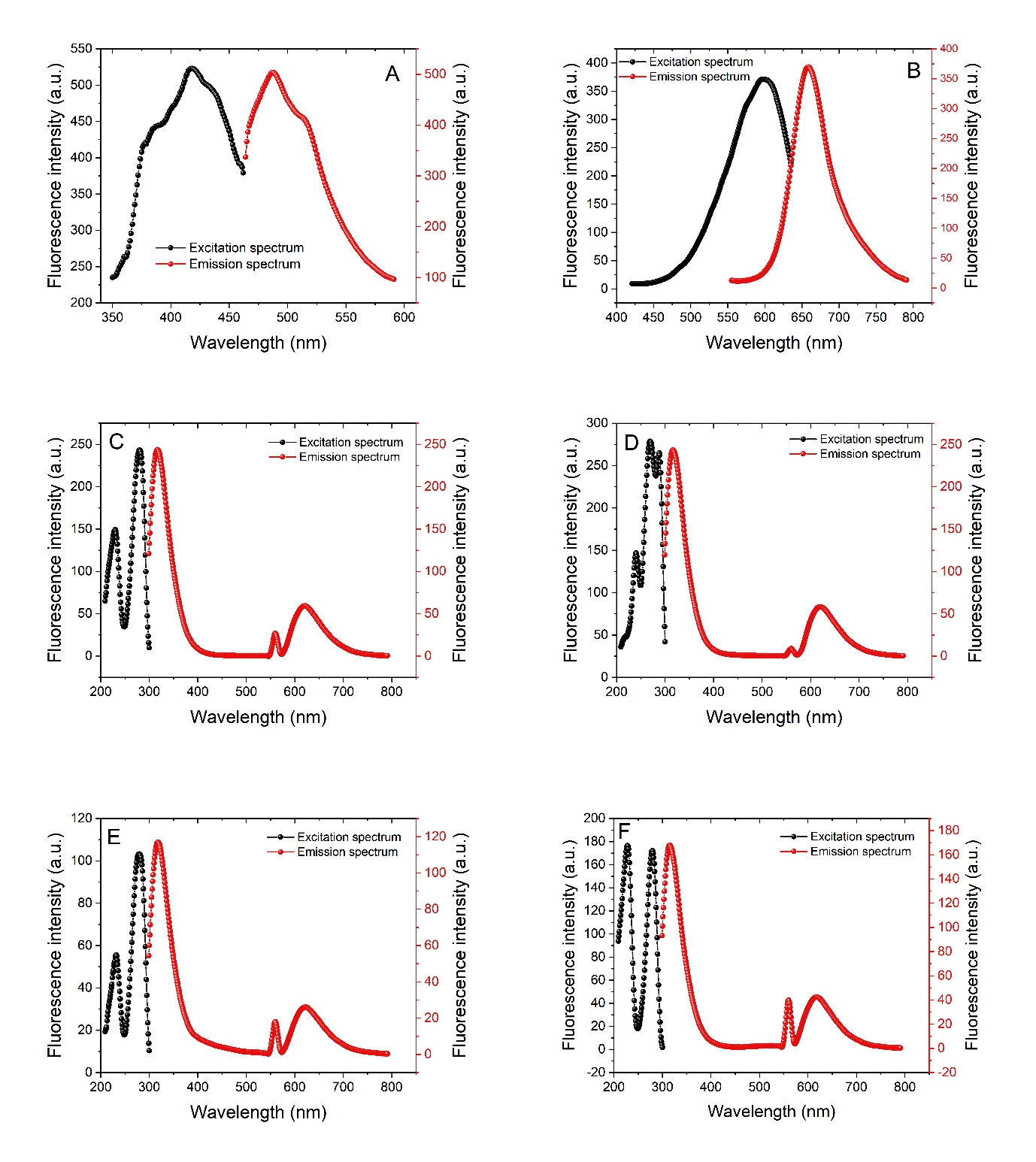


**Figure S1.** Excitation and emission spectra of the ThT (A), NR (B), Dopamine (C), Epinephrine (D), (E) DOPAL, and Levodopa (F).

**
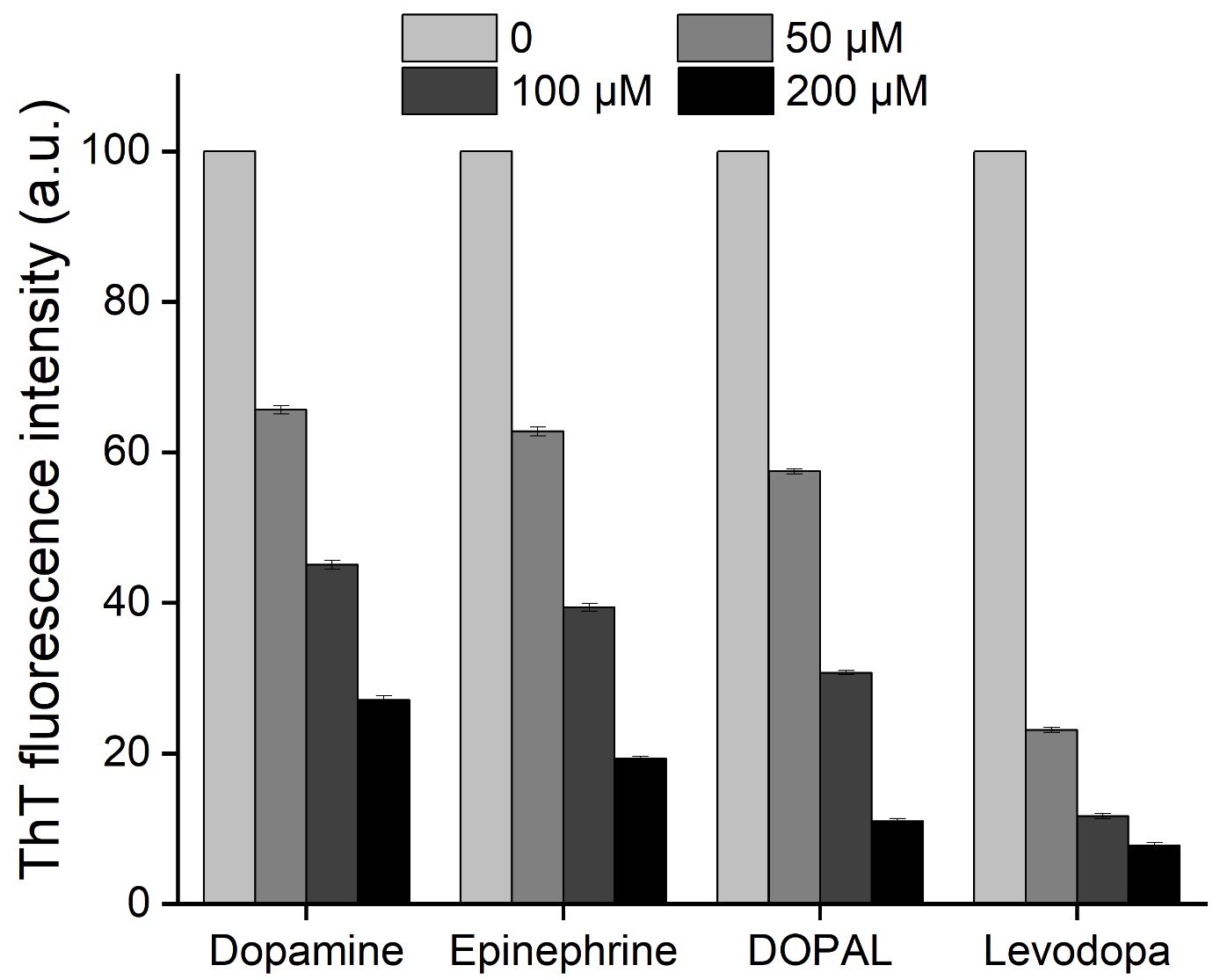
**

**Figure S2.** ThT fluorescence intensity of protein samples incubated in the presence of increasing concentrations of catechols for 60 h under amyloidogenic conditions. Data are expressed as mean ± SD with n=3.


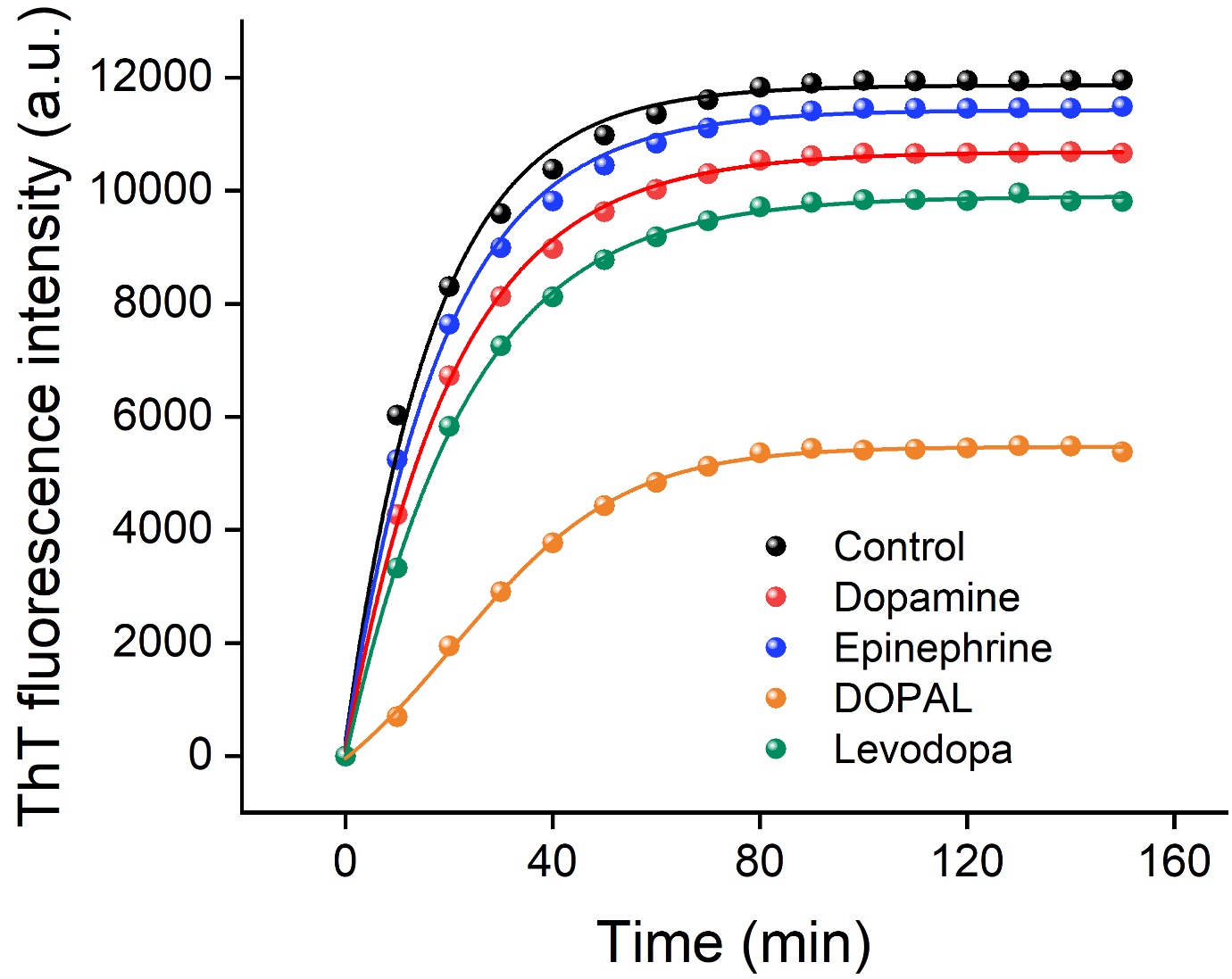


**Figure S3** Effect of catechols (200 μM) on the secondary nucleation of α-syn fibrillization. Data are expressed as mean ± SD with n=3.

**
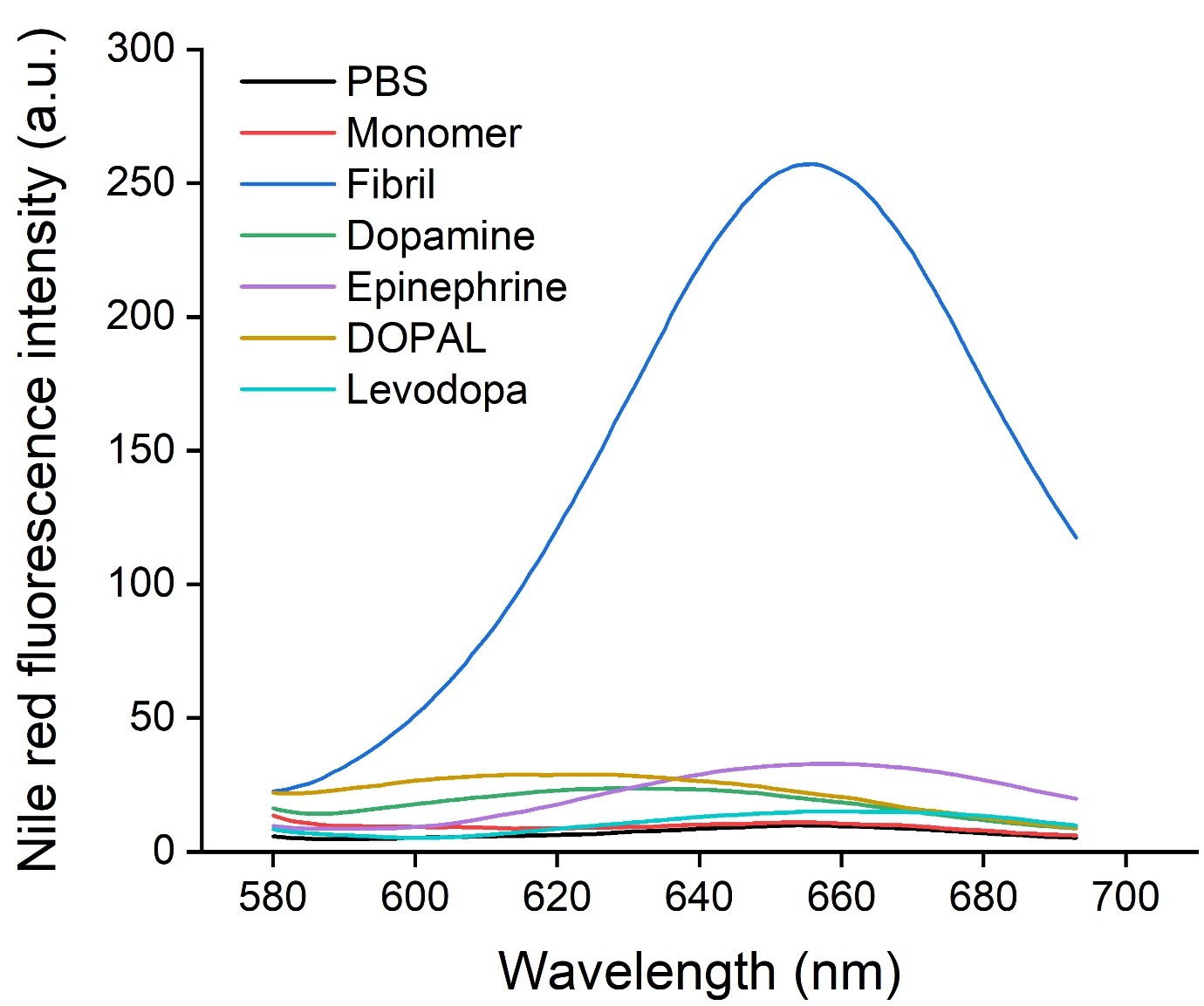
**

**Figure S4.** Nile red fluorescence intensity of protein samples incubated in the presence of 200 µm catechols for 60 h under amyloidogenic conditions. Data are expressed as mean ± SD with n=3.


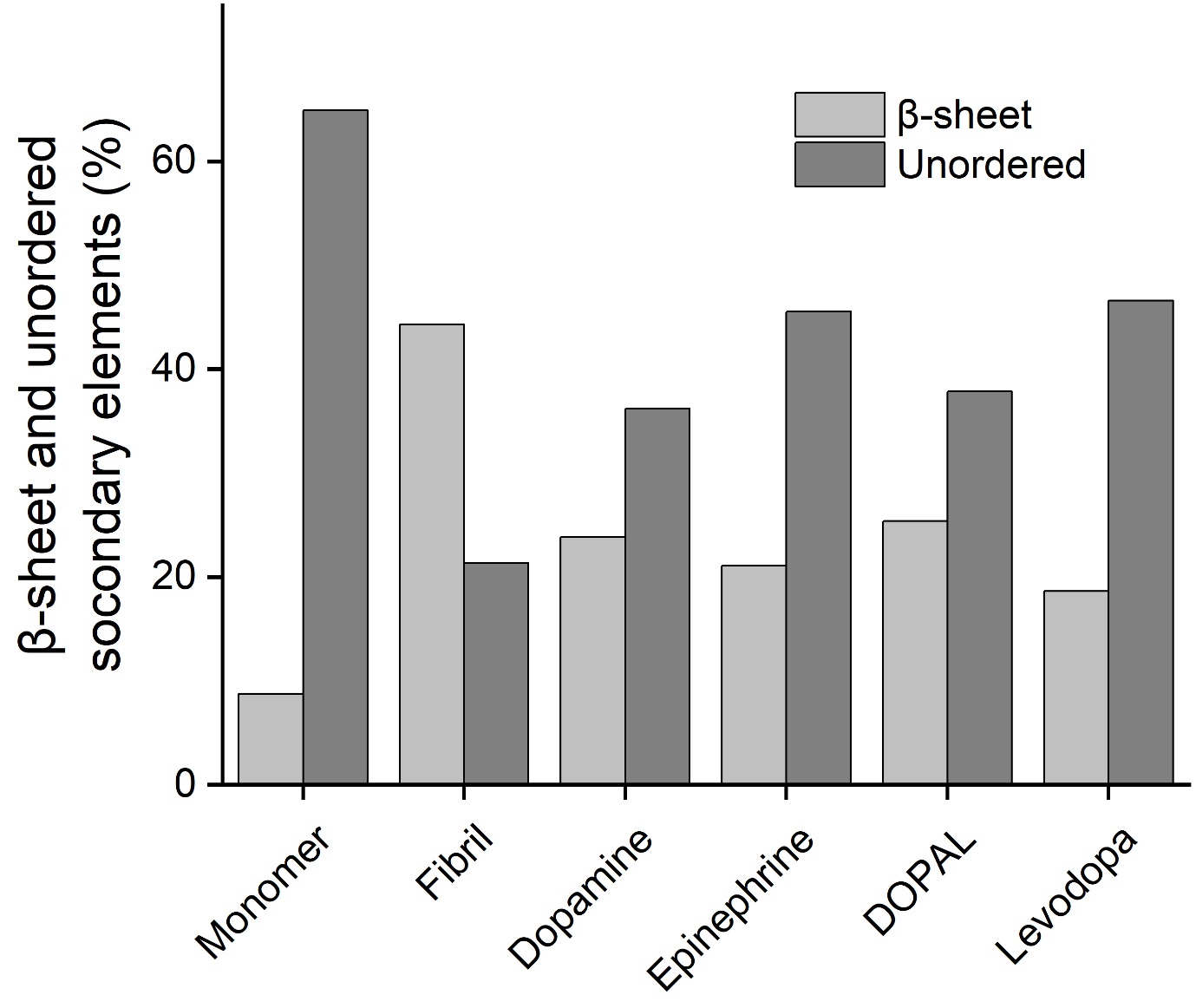


**Figure S5.** Changes in the secondary structural elements of α-syn induced by 200 µm catechols, as calculated from far-UV CD spectra.

**
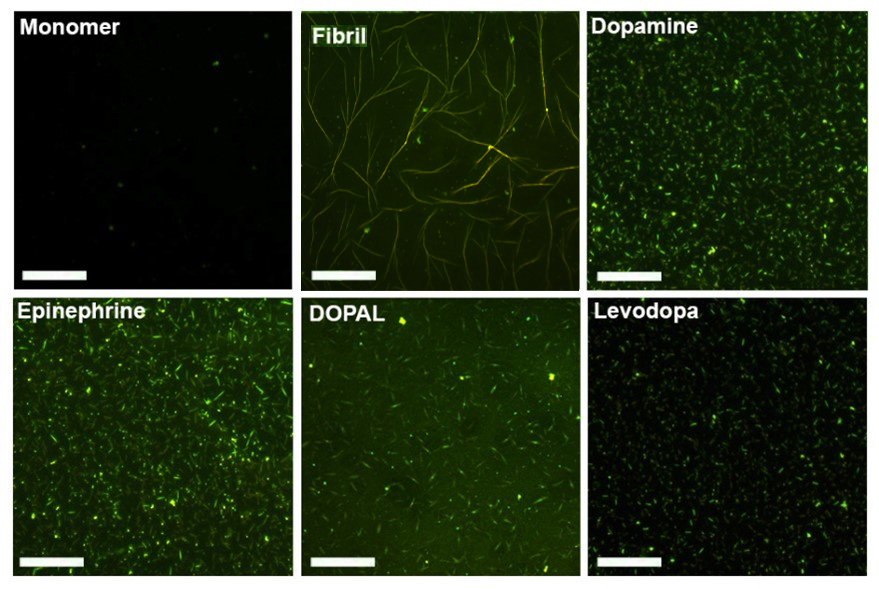
**

**Figure S6.** Fluorescence microscopy images of protein samples incubated alone or in the presence of 200 µm catechols for 60 h. The scale bar represents 100 μm.


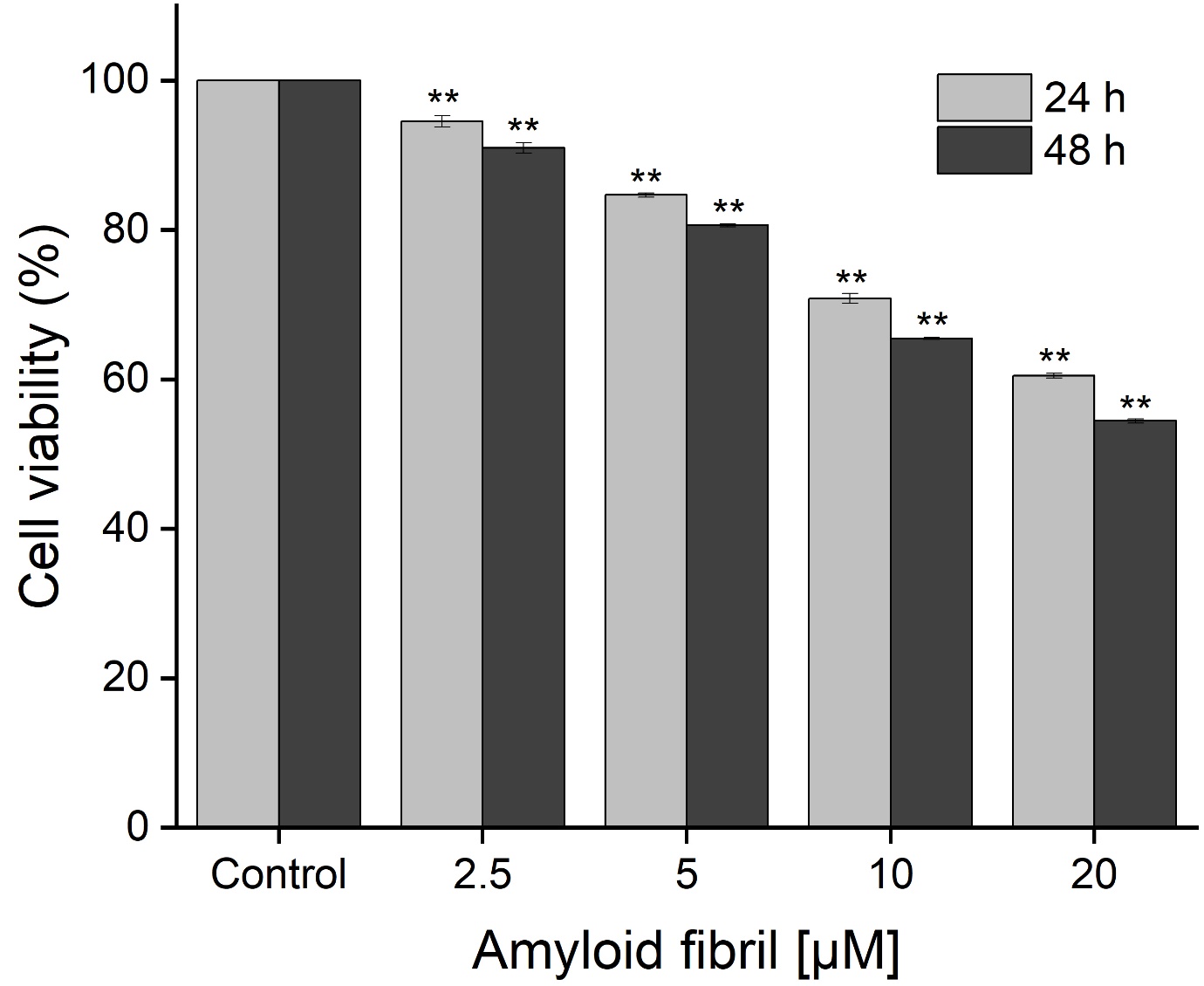


**Figure S7.** Dose-dependence cytotoxicity of α-syn fibrils. SH-SY5Y cells were incubated with different concentrations (0–20 μM) of α-syn fibrils for 24 h and cell viability was evaluated by the MTT reduction assay. The data were expressed as percentage of cell viability in untreated control cells and each value represents the mean ± SD (n=5). ^**^p < 0.01, significantly different from control cells.


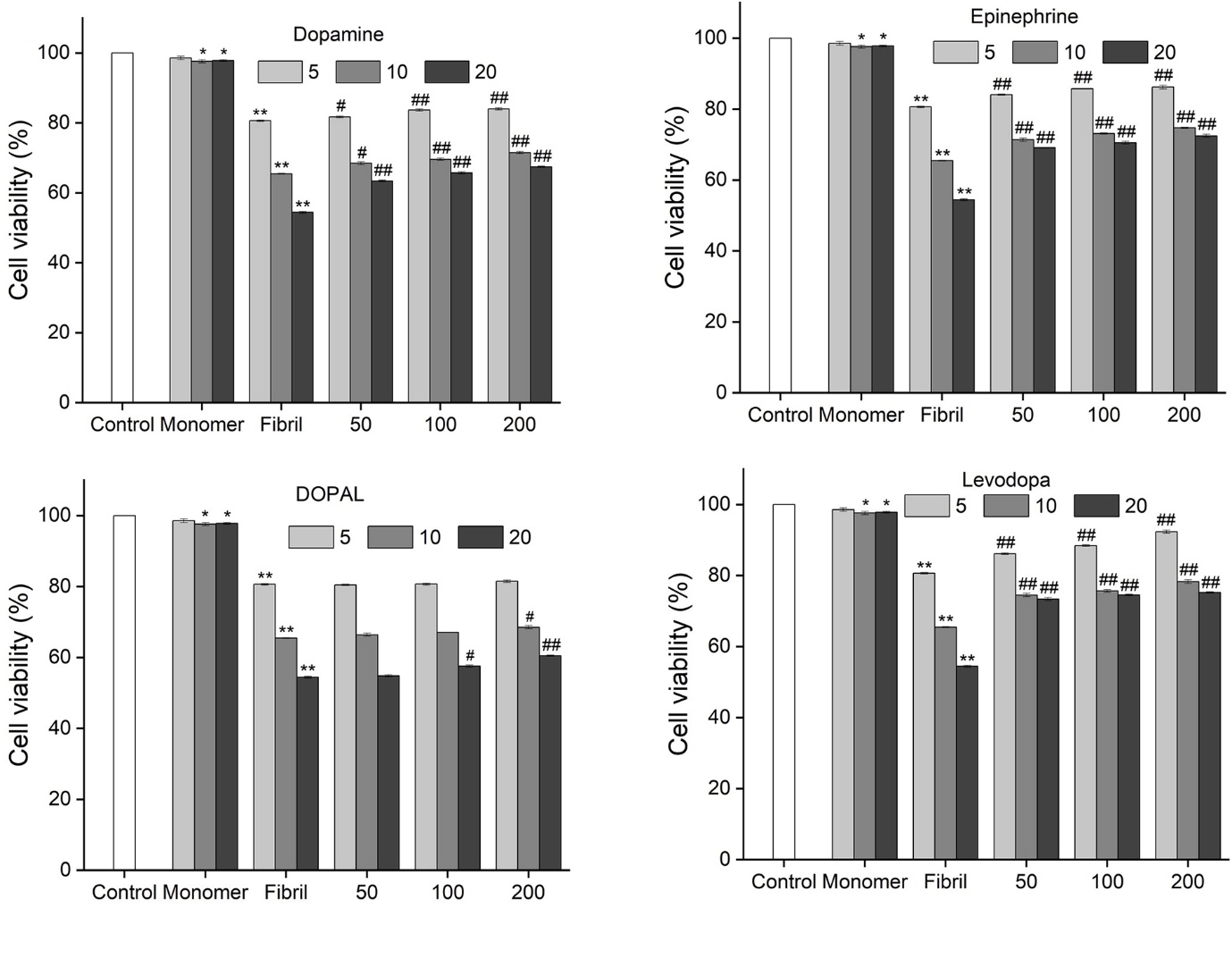


**Figure S8.** Cytotoxicity evaluation of various concentrations of α-syn aggregates produced in the absence or presence of increasing concentrations of catechols. Data are expressed as mean ± SD with n=5.^#^p < 0.05, ^##^p < 0.01, signiﬁcantly different from cells exposed only to α-syn specified amyloid ﬁbrils.


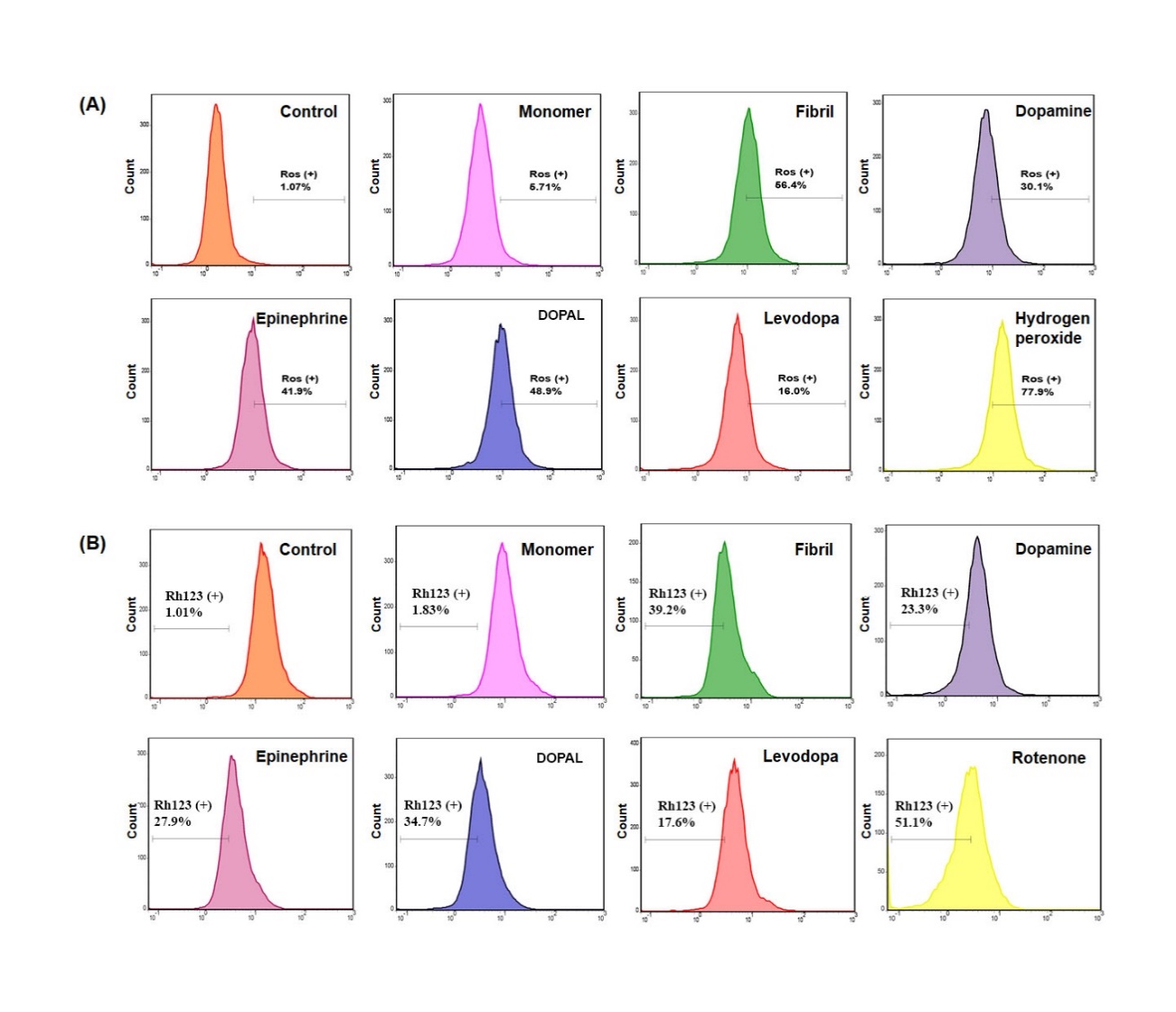
 **Figure S9.** The effect of catechols on the α-syn aggregates-induced intracellular ROS and mitochondrial membrane potential in SH-SY5Y cells was evaluated by flow cytometry. A and B are Quantitative histograms of the mean fluorescence intensity of DCFDA and Rhodamine 123 probes expressed relative to the level of control cells, respectively. Data are expressed as mean ± SD with n=3.

.


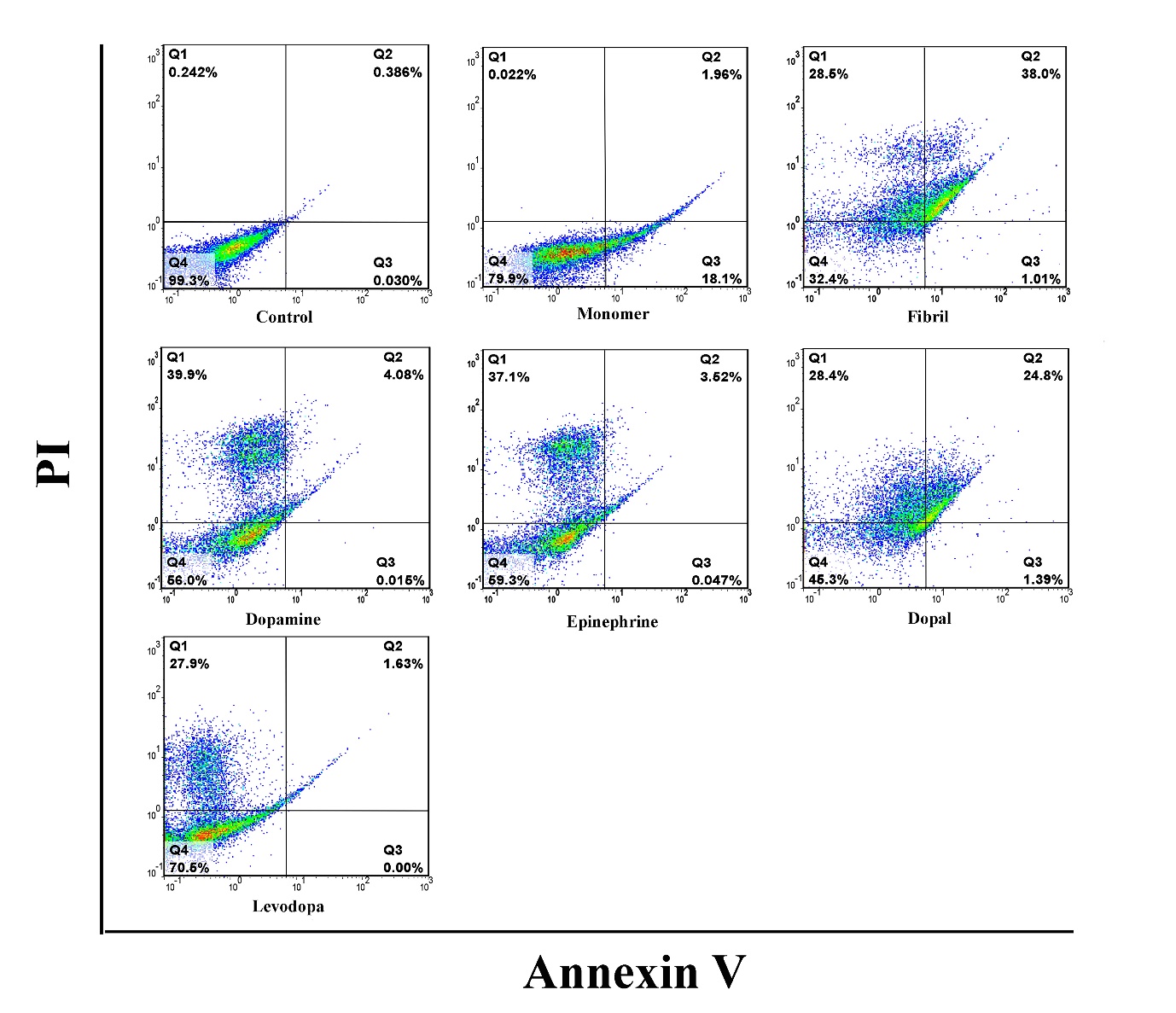


**Figure S10.** Flow cytometric analyses of apoptosis and necrosis in SH-SY5Y cells exposed to 20 µM α-syn aggregates, produced in the absence or presence of 200 µM. Data are expressed as mean ± SD with n=3.

*.*


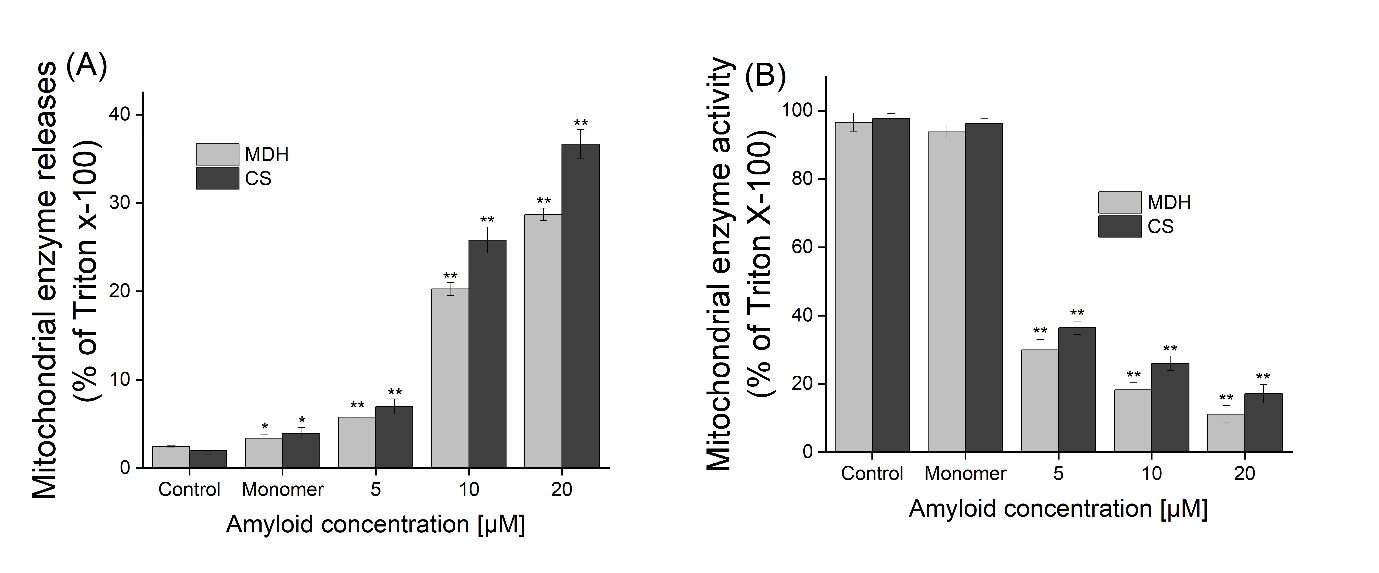


**Figure S11.** Dependency of mitochondrial enzyme release (A) and enzyme activity inhibition (B) on α-syn aggregates concentrations. Data are expressed as mean ± SD with n=3. ^*^p < 0.05, ^**^p < 0.01, signiﬁcantly different from control.
